# Neuroimmunomodulatory Properties of Flavonoids and Derivates: A Potential Action as Adjuvants for the Treatment of Glioblastoma

**DOI:** 10.3390/pharmaceutics14010116

**Published:** 2022-01-04

**Authors:** Ravena Pereira do Nascimento, Balbino Lino dos Santos, Jéssika Alves Oliveira Amparo, Janaina Ribeiro Pereira Soares, Karina Costa da Silva, Monique Reis Santana, Áurea Maria Alves Nunes Almeida, Victor Diógenes Amaral da Silva, Maria de Fátima Dias Costa, Henning Ulrich, Vivaldo Moura-Neto, Giselle Pinto de Faria Lopes, Silvia Lima Costa

**Affiliations:** 1 Laboratory of Neurochemistry and Cell Biology, Department of Biochemistry and Biophysics, Institute of Health Sciences, Federal University of Bahia, Salvador 40110-902, Bahia, Brazil; ravenanascimento@ufba.br (R.P.d.N.); balbino.lino@univasf.edu.br (B.L.d.S.); amparojessikaufba@gmail.com (J.A.O.A.); janainaribeirolabnq@gmail.com (J.R.P.S.); karinacostads@gmail.com (K.C.d.S.); moniquereisant@gmail.com (M.R.S.); aurea.maria.almeida@gmail.com (Á.M.A.N.A.); vdsilva@ufba.br (V.D.A.d.S.); fatima@ufba.br (M.d.F.D.C.); 2 Academic College of Nurse, Department of Health, Federal University of Vale do São Francisco, Petrolina 56304-205, Pernambuco, Brazil; 3 National Institute for Translational Neurosciences (INCT/CNPq INNT), Rio de Janeiro 21941-902, Rio de Janeiro, Brazil; vivaldomouraneto@gmail.com; 4 Department of Biochemistry, Institute of Chemistry, University of São Paulo, São Paulo 05508-000, São Paulo, Brazil; 5 Institute of Biomedical Sciences, Federal University of Rio de Janeiro, Rio de Janeiro 21941-902, Rio de Janeiro, Brazil; 6 Paulo Niemeyer State Institute of the Brain, Rio de Janeiro 20230-024, Rio de Janeiro, Brazil; 7 Department of Marine Biotechnology, Admiral Paulo Moreira Institute for Sea Studies (IEAPM), Arraial do Cabo 28930-000, Rio de Janeiro, Brazil; giselle.faria@gmail.com

**Keywords:** glioblastomas, tumor microenvironment, microglia, flavonoids, cytokines, miRNAs

## Abstract

Glioblastomas (GBMs) are tumors that have a high ability to migrate, invade and proliferate in the healthy tissue, what greatly impairs their treatment. These characteristics are associated with the complex microenvironment, formed by the perivascular niche, which is also composed of several stromal cells including astrocytes, microglia, fibroblasts, pericytes and endothelial cells, supporting tumor progression. Further microglia and macrophages associated with GBMs infiltrate the tumor. These innate immune cells are meant to participate in tumor surveillance and eradication, but they become compromised by GBM cells and exploited in the process. In this review we discuss the context of the GBM microenvironment together with the actions of flavonoids, which have attracted scientific attention due to their pharmacological properties as possible anti-tumor agents. Flavonoids act on a variety of signaling pathways, counteracting the invasion process. Luteolin and rutin inhibit NFκB activation, reducing IL-6 production. Fisetin promotes tumor apoptosis, while inhibiting ADAM expression, reducing invasion. Naringenin reduces tumor invasion by down-regulating metalloproteinases expression. Apigenin and rutin induce apoptosis in C6 cells increasing TNFα, while decreasing IL-10 production, denoting a shift from the immunosuppressive Th2 to the Th1 profile. Overall, flavonoids should be further exploited for glioma therapy.

## 1. Introduction

Glioblastomas (GBMs) are high-grade malignant brain tumors classified as grade IV by the World Health Organization (WHO). They are the most common and devastating primary malignant brain tumor of the central nervous system (CNS) in adults. GBMs are highly infiltrative and morphologically very heterogeneous, characterized by anaplasia, intense mitotic activity, as well as the presence of necrosis and microvascular proliferation [1]. Currently, the protocol adopted for the GBM treatment is based on surgery followed by radiation therapy and chemotherapy. However, despite recent advances in therapy, the average survival time for patients is still around 14 months [2].

In the physiopathology of GBMs and other diffuse tumors at present is the complexity of the cell population in the tumor parenchyma and its microenvironmental interactions needs to be better understood. Its interaction with healthy cells, as microglia, involves regulation of immunologic responsiveness, which is related to tumoral aggressiveness and invasion. M2-polarization of microglia involve immunosuppressive and tumor-supportive properties, exhibiting reduced phagocytic activity [3]. Therefore, exogenous compounds that demonstrated antitumor activity and that influenced the immunomodulatory profile in the tumor niche may be key allies to improve the conventional treatment for solid tumors, such as GBMs. The search for alternative drugs for therapeutic interventions against brain tumors has shed light on phytochemicals, including plant-based drugs [4], and flavonoids, a group of molecules that have raised interest in this regard. Flavonoids are products of the secondary metabolism of plants, present in almost all fruits and vegetables [5]. Studies conducted over the last two decades, and especially in the last years, have demonstrated the antitumor potential of the flavonoids both in in vitro and in in vivo models of GBMs [5,6,7,8]. Moreover, these compounds also showed the potential to modulate the inflammatory response of microglia/macrophages associated with tumorigenesis inhibition [9,10,11]. The present review discusses the main aspects of GBM physiopathology and advances in the interaction between tumor and microenvironment. The anti-glioma effects of different flavonoids and their derivatives are updated in our manuscript. Further flavonoid-promoted anti-inflammatory effects on CNS-resident and invading immune cells are also discussed as a new concept for GBM-adjuvant treatment based on immunomodulation.

## 2. Glioblastoma: Origin, Histology and Classification

**Figure 1:**
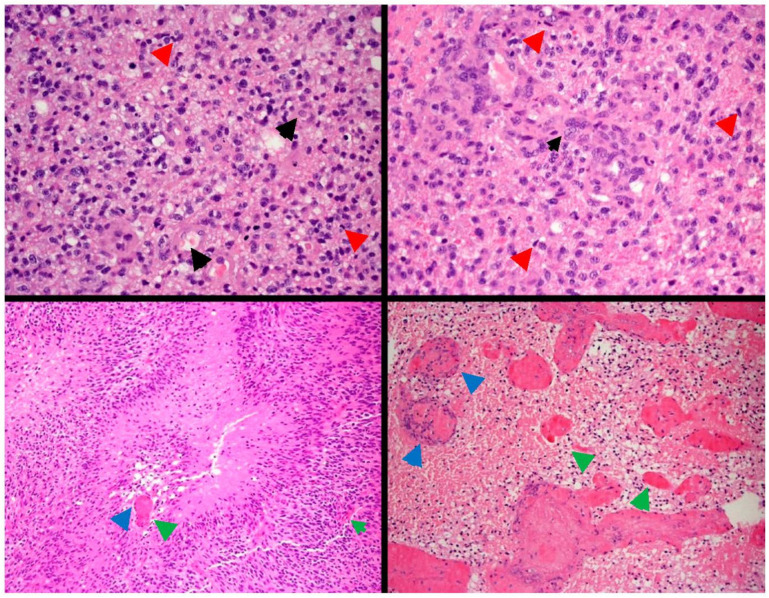
Glioblastoma with a poor prognosis based on its immunohistochemistry panel. Paraffin sections show fragments of a densely hypercellular glial tumor. The tumor is composed predominantly of moderately pleomorphic fibrillary astrocytes arranged in diffuse sheets. A small component of gemistocytic cells is noted (black arrows). There are moderate numbers of mitotic figures (red arrows). Foci of microvascular proliferation with multilayering of atypical cells around vessel lumena are also noted. Prominent pallisading and confluent necrosis is noted (blue arrows). Several of the latter areas incorporate thin-walled necrotic blood vessels (green arrows). The features are of glioblastoma multiforme (WHO Grade IV). GFAP: positive; Nestin; positive (high); IDH-1; R132H: negative (not mutated); ATRX: positive (not mutated); MGMT: negative (likely methylated); p53: positive p16; CDKN2A: negative; Topoisomerase labeling index: Approximately 35%. Case courtesy of RMH Neuropathology, Radiopaedia.org, rID 41309, modified.

Glioblastoma is the most common and aggressive CNS tumor, which usually appears in the cerebral hemispheres of adults [1,12]. Despite advances in neurosurgery, radiotherapy, and chemotherapy, it is one of the most difficult neoplasms to treat, with an average overall survival of 12 to 15 months [13,14]. This short survival rate is attributed to clinical limitations, such as advanced tumor progressionat the time of diagnosis, and molecular characteristics as the heterogeneous and invasive nature of GBM cells (Figure 1), resulting in high recurrence rates [1,14,15].

Over the past decade, progress in genomic cancer studies has provided relevant information regarding the mechanisms of tumorigenesis and genetic mutations in primary and secondary GBMs [16,17]. Due to its remarkable histological variability, an important update of the WHO classification for CNS tumors in 2016 highlighted molecular changes and differences in patterns of gene expression with diverse mutations that serve as determinants in different types of cancer [1,18]. Evidence obtained from genetic and molecular analysis indicates that primary and secondary GBMs are distinct entities of the disease that develop through different molecular pathways, exhibit different transcriptional patterns and recurrent gene mutations [13]. Typical genetic changes in primary GBMs include overexpression of the epidermal growth factor receptor (EGFR), mutations in the tumor suppressor protein phosphatase and tensin homolog (PTEN) and the telomerase reverse transcriptase (TERT) promoter, as well as the loss of chromosome 10q. On the other hand, secondary GBMs often show mutations of isocitrate dehydrogenase 1 and 2 (IDH1/2), the pro-apoptotic 53 kDa tumor protein (p53 or TP53), and the Thalassemia Alpha/Mental Retardation X-linked gene (ATRX), as well as the loss of chromosome 19q [2,13,16,18]. In addition, these two types of GBMs are notably different from each other in terms of methylation patterns, cell signaling pathways and patterns of matrix metalloproteinases (MMPs) activation 19q [16,19,20].

According to the Atlas of the Cancer Genome (TCGA), the GBM subtypes are: proneural, neural, mesenchymal and classic. Proneural GBMs have the lowest occurrence rate, develop mainly in younger patients with secondary GBM and are characterized by changes in platelet-derived growth factor receptor A (PDGFRA) and mutations in IDH1 and TP53 [21,22]. The neural subtypes are characterized by the expression of neural markers and high EGFR expression, which is similar to those of normal brain tissues. The high frequency of neurofibromatosis 1 (NF1) mutations and the overexpression of chitinase-3-like protein 1 (CHI3L1) are predominant characteristics of the mesenchymal subtype. In addition, mutations in genes involved in the kβ nuclear factor pathways (NF-kβ), overexpression of the vascular endothelial growth factor (VEGF) and the platelet and endothelial cell adhesion molecule 1 (PECAM1) are identified. Moreover, in the classic GBM, the predominance of EGFR mutations, cyclin-dependent kinase inhibitor 2A (CDKN2A) deletion, and the lack of TP53 mutations are observed. It is suggested that a better prognosis is associated with the proneural GBM in comparison to the other subtypes 19q [16,22,23]. 

Although there are great advances in the identification of genetic and molecular markers, the mechanisms underlying the origin of GBMs are not fully understood [24]. There are many theories about their origin. The two most discussed and dominant are the dedifferentiation theory and the stem cell theory. The stem cell theory suggests that the neural stem cells (NSC), or oligodendrocyte progenitor cells, are the cells of origin. Meanwhile, the dedifferentiation theory suggests that differentiated mature astrocytes, ependymal cells and oligodendrocytes could be the precursors of GBM [17,25,26]. 

Due to their self-renewable and multipotent potential, the NSCs, which give rise to neuronal and glial progenitor cells, undergo combinations of specific genetic alterations, as in tumor suppressor genes (PTEN, Ink4a/Arf, Nf1, p53 and Rb1) and oncogenes (EGFR, kRas, Akt, PDGF and IDH1 ligands R132H), showing signs of neoplasia [24,27]. Thus, because they exhibit similar characteristics to those of GBMs, such as the activation of developmental signaling pathways, motility and the association with the basement membranes of blood vessels, NSC are strong candidates as cells of origin of GBMs [17,24,28]. 

Once GBMs are characterized as heterogeneous tumors due to the presence of different subpopulations in the tumor mass, the bulk could have neoplastic cells of different origins [29]. Different mature CNS cell types have specific mutations that lead to specific subtypes of the tumor [21]. Different subpopulations of astrocytes submitted to the same oncogenic mutation can rise to different types of gliomas [30]. The combined activation of Ras and AKT or deletions in PTEN, TP, P53, NF-1 and RB -1 demonstrated gliomagenesis failures [24]. In contrast, transient receptor potential (TRP) induced mutations in astrocyte glial fibrillary acidic protein (GFAP) and glutamate aspartate transporter (GLAST) showed significant low-grade astrocytoma formation with differences in growth and progression kinetics. Due to the tumor heterogeneity, CNS cell types of variable origins is responsible for the variety of molecular subtypes of GBMs [30].

## 3. Glioblastoma Microenvironment

**Figure 2:**
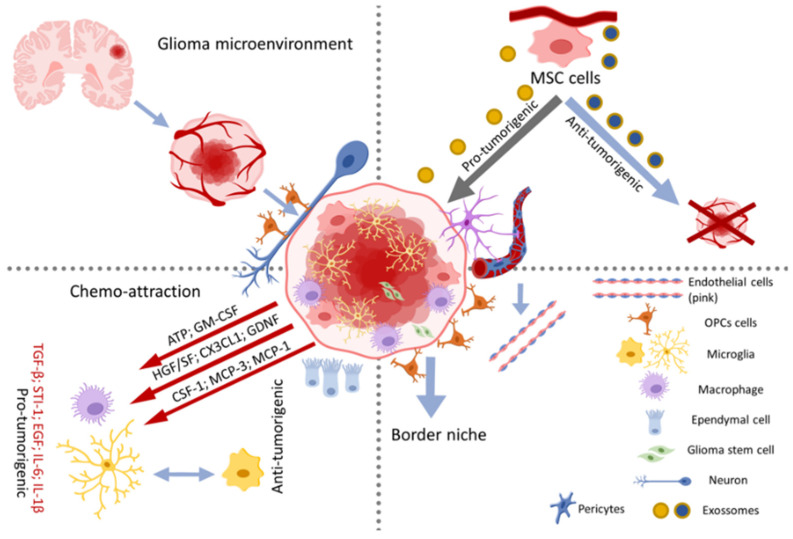
Glioma microenvironment. Interactions between healthy CNS cells and the perivascular system within the glioma. The tumor microenvironment is rich in tumor stem cells responsible for maintaining tumor progression and transformed glial cells (such as astrocytes and microglia), as well as other cells responsible for maintaining tumor angiogenesis (such as mesenchymal and perivascular cells). Tumor cells physically and chemically interact with other cells, such as neurons that impact the activity and functions of these cells. In the perivascular system, mesenchymal cells (MSCs) are recruited and release the content of pro-tumorigenic or antitumorigenic exosomes. In addition, pericytes are recruited for neovascularization in favor of the tumor. The macrophages residing in the CNS, the microglia, undergo chemoattraction and start to release pro-tumorigenic factors that help in the maintenance of the tumor. In addition, at the edge of the mass, GBM cells use oligodendrocyte progenitor cells (OPCs) and microglia to acquire characteristics such as stem cells, favoring tumor invasion. Chemo attraction factors: Granulocyte-Macrophage Colony-Stimulating Factor (GM-CSF), Fractalkine or chemokine (C-X3-C motif) Ligand 1 (CX3CL-1), Glial Cell Line-Derived Neurotrophic Factor (GDNF), Colony-Stimulating Factor 1 (CSF-1), Monocyte Chemoattractant Protein-1 (MCP-1) and (MCP-3).

Interactions between astrocytes, neurons, microglia, oligodendrocytes, mesenchymal stem cells (MSCs), neural stem cells (NSCs), lymphocytes and ependymal cells maintain cerebral homeostasis. The communication between healthy cells and tumoral cells is important for establishing the tumor microenvironment [31]. The cellular complexity of high-grade glial tumors is the result of the non-glial cell type recruitment into the tumor, contributing to the complexity of the oncological target structure [32] (Figure 2).

### 3.1. Glioblastoma Interactions with Glial Cells and Neurons

GBMs directly interact with their microenvironment in the CNS, especially with a perivascular niche, also composed of several stromal cells including astrocytes, microglia, fibroblasts, pericytes and endothelial cells, supporting tumor progression [33]. Astrocytes have different roles in the CNS. They are responsible for the regular homeostasis of the CNS, supporting neurons through neurotransmitter precursor release. Besides, they regulate metabolism, release diverse chemical substances, contribute to functional hyperemia and regulate blood-brain [31,34]. Surrounding GBM cells, astrocytes respond with a reactive phenotype characterized by GFAP overexpression, and the crosstalk to other components of the environment is poorly understood [35]. Reactive astrocytes aid the parenchymal infiltrative capacity of glioma cells and stimulate their uncontrolled proliferation by expressing matrix metalloproteinase-2 (MMP2) and secreting stromal cell-derived factor-1 (SDF1) [36]. In this context, reactive astrocytes secreted factors that regulate glioma cell invasion as the receptor activator of NFκB ligand (RANKL) [37].

Other important aspects of GBM/astrocyte communication, are direct cell-cell contacts through communicating junctions, ion channels, microtubules and tunneling nanotubes [36,38] and indirect interactions through the secretion of molecules, the release of gliotransmitters and extracellular vesicles (EVs), which stimulate healthy astrocytes to internalize them and to acquire a tumor support phenotype via p53 and MYC signaling pathways [39]. EVs are responsible to manipulate the GBM microenvironment, also exporting microRNAs (miR), like miR-21 and miR-451, which are uptaken by microglia and monocytes/macrophages promoting their proliferation [40]. MicroRNAs or exosomal microRNAs (exomiRs) are the class of wide-spread short non-coding RNAs that inhibit translation via binding to the mRNA of target genes, and are responsible to regulate the proliferation, migration and invasion of glioma cells [41]. Also, miRs are involved in the progress and development of gliomas and are overexpressed like miR-96 and miR 1290 associated with the metastasis of glioma [42].

Microglia cells are the mononuclear macrophages resident in the CNS and comprise 5 to 20% of all glial cells in the CNS [43]. As the brain parenchyma’s resident immune cells, microglia act as central communicators between the nervous system and the immune system, as they are the first sentinels to protect against invading pathogens and tissue damage [44]. Microglia and macrophages associated with GBMs infiltrate the tumor center by chemoattraction. These innate immune cells are meant to participate in tumor surveillance and eradication, but they become compromised by GBM cells and exploited in the process [45]. In brain cancers, these macrophage populations infiltrate the tumor area and contribute up to 50% of the total non-neoplastic cells [46]. This has implications for therapy, since drugs that penetrate the CNS to reach microglia, as well as drugs with peripheral action affecting monocytes are needed, for combatting GBMs. Microglia/macrophages, and their pro- and anti-inflammatory subgroups, may have different functions in the biology of GBMs at specific stages of tumor evolution [45]. It seems that modulation of the inflammatory response via microglia affects both the maintenance of tumors and their depletion. Microglial cells and macrophages demonstrated influence in the effectiveness (positively or negatively) of many treatments for GBMs, including chemotherapy, radiotherapy, virotherapy and immunotherapy [46]. Activation of microglia and the subsequent neuroinflammation modulated by interleukin-6 seem to be involved in blood-brain barrier (BBB) dysfunction, commonly observed in several CNS diseases, including brain tumors [47].

Oligodendrocytes are considered as stroma cells for diffuse glioma, especially GBM. There is an up-regulation of the invasive activity of GBM cells via the Angiopoietin-2 (Ang-2) signaling pathway [34]. Also, oligodendrocyte progenitor cells (OPCs) “in the border niche” may induce stemness and chemo-radioresistance in GBM cells, providing a supportive function for promoting GBM aggressiveness. In this context, neuronal activity induces proliferation of both OPCs and GBM cells. GBM cells prefer to migrate within the fasciculus of axons where abundant oligodendroglial-like cells (OLCs) are found, including OPCs, particularly at the border [48].

There are significant interactions between GBMs and neurons, which are responsible for symptoms in patients. Clinical observations of patients with GBM having epileptic episodes with impaired memory were reported, among other effects associated with neuron dysfunction. The effect of the tumor on neural networks has been proposed due to mechanical pressure imposed by the tumor mass. However, considering the variety of factors released by the glioma and the complexity of the intercellular signaling mechanisms, the effects are probably much more complex [48]. In a study developed by Portela and colleagues [49], GBM activates the WNT pathway in neurons by displaying a network of tumoral microtubes involving neurons and causing Wg Frizzled1 (Fz1) receptor accumulation. Thus, the results are Wg depletion and neurodegeneration of neurons, favoring proliferation and infiltration of GBM cells due to the extinction of the Wg signaling.

There are also neurotoxic effects in the tumor environment due to the exacerbated release of the neurotransmitter glutamate by neurons. GBMs release extracellular glutamate promoting tumor growth and tissue invasion, due to the activation of glutamate receptors in the glioma cell itself and the activation of glutamate receptors in neighboring healthy neurons [50]. Both metabotropic and ionotropic glutamate might act as a growth factor and as a signal mediator in both autocrine and paracrine signaling [51,52]. Glutamate promotes tumor cell invasion, inducing intracellular Ca^2+^ oscillations through the activation of Ca^2+^ permeable AMPA receptors [53]. This mechanism is remarkably similar to that used by migratory neurons during the development of the cerebellum, during which Ca^2+^ oscillations mediated by NMDA receptors guide the migration of granular cells [54]. The tumoral microenvironment shows an extraordinarily complex interaction with different mature neural cells. On the other hand, the intratumor heterogeneity depends on the presence of stem cells (SC) [55].

Flavonoids seem to modulate the interaction between glioma and glial cells, contributing to the non-maintenance of the tumor without damaging healthy nervous cells. A study developed by Das et al. [56], demonstrated that flavonoids activated caspases for apoptosis induction in human glioblastoma T98G and U87MG cells but not in human normal astrocytes. Further, Santos et al. [5] investigated the effects of several polyhydroxylated flavonoids namely, rutin, quercetin (F7), apigenin (F32), chrysin (F11), kaempferol (F12) and 3′,4′-dihydroxyflavone (F2) in human GL-15 glioblastoma cells. They observed that all flavonoids decreased the number of viable cells and the mitochondrial metabolism. In vivo studies, with different flavonoids, have already demonstrated the ability of these compounds to suppress growth of the glioma cell population, without compromising the healthy cells around the tumor. For example, Guo et al. [57] demonstrated that Ampelopsin inhibited tumor growth and progression in mouse xenograft models, and induced apoptotic response via both intrinsic and extrinsic signaling pathways. Aroui et al. [58] showed that narigenin inhibited invasion and angiogenesis in an in vivo model of subcutaneous glioma. Further, in a prospective study of dietary flavonoid intake and risk of glioma in US citizens, Bever et al. [59] concluded that increased dietary intakes of flavan-3-ol and polymeric flavonoids were associated with decreased risks of developing this brain tumor, without compromising the functions of other CNS cells.

### 3.2. Properties of Cancer Stem Cells, MSCs and Further Stem Cells in Glioma Heterogeneity

Considering the two hypotheses about the origin of GBM heterogeneity, cancer stem cells (CSCs) self-renew and initiate the formation of tumors. CSCs give rise to phenotypically diverse cancer cells and reside in specialized niches, where the interaction with the microenvironment regulates cellular behavior [60]. According to Pavon et al. [61], CD133+ GBM cells express molecular signatures of MSCs, NSCs and pluripotent SCs, thus possibly enabling differentiation into both neural and mesodermal cell types.

Glioma stem cells (GSCsor CSCs) could differentiate to endothelial cells inducing new vessels via a phenomenon known as vascular mimicry. However, that ability does not lead to the form of mature and proper blood vessels which would counteract hypoxia [62]. GSCs occur in perivascular and perinecrotic niches expressing stemness biomarkers. The perivascular niches are represented by endothelial cells associated with Nestin+ and CD133+ cells, which condition angiogenesis and tumor growth [63]. Blood vessels support GSCs; these tumor cells in turn may regulate and contribute to the tumor vasculature, by directly transdifferentiating into endothelial cells or through the secretion of regulatory growth factors such as VEGF and hepatoma-derived growth factor (HDGF) [64].

MSCs are stromal cells capable of self-renewal and differentiating into multiple phenotypes [65]. These cells can be isolated from a variety of tissues, such as, for example, umbilical cord [65], bone marrow [66] and adipose tissue [67]. There is growing evidence that MSCs may be located outside the vasculature and that perivascular cells, often called “pericytes”, may include MSCs, suggesting that MSCs located around blood vessels may migrate to injured tissue and that intravenous or intradermal administration of MSCs improves healing and reduces inflammatory infiltrate [68]. In GBMs, these cells appear to support cellular growth, as shown in vitro and in vivo [69]. MSCs also produce exosomes, which can induce the activation of macrophages/microglia and polarize microglia towards M2 phenotypes, inhibiting the release of pro-inflammatory cytokines and promoting tissue repair and nerve regeneration in mechanical brain trauma and neurodegenerative diseases [70,71]. Moreover, exosomes released by MSCs increase proliferation and clonogenicity of tumor-initiating GSCs through micro RNAs, such as miR1587 [72].

There is experimental evidence that MSCs have a dubious role in modulating the environment of malignant tumors. Tumor inhibition mediated by MSCs is induced by the suppression of angiogenesis, regulation of signaling pathways and promotion of apoptosis in tumor microenvironments [73], including GBMs [74,75]. On the other hand, MSCs show tropism for tumor cells, which favors the tumor microenvironment, by releasing pro-tumorigenic factors (secretome) [76], in addition to the crosstalk between tumor cells and MSCs, which increases the metastatic potential and promotes epithelial-to-mesenchymal transition [77]. Also, MSCs can be modified to produce miR involved in suppressing the proliferation of gliomas, such as miR133b [78], stimulating the senescence of glioma cells by miR34a [71], thus constituting a possible therapeutic tool against gliomas.

There are few studies demonstrating the effect of flavonoids on mesenchymal and glioma cells simultaneously. In studies with other cancer cells, flavonoids like epigallocathechin gallate (EGCG), a flavan-3-ol, induced apoptosis significantly and inhibited colony formation and cell migration in nasopharyngeal carcinoma (NPC) CSCs, including the sphere-derived NPC TW01 and TW06 cell lines Amawi et al. [79]. In a study developed by Nascimento et al. [80], agathisflavone induced dose-dependent toxicity in GL-15 and U373 human GBM cells and was not toxic for human MSCs, but modified their pattern of interactions with GBM cells in co-culture, promoting mechanisms that may contribute to the death or maintenance of the tumor.

In this context, much needs to be understood about the mechanisms involved between glioma cells and other CNS cells as well as the mechanisms involved in the maintenance of the GBMs microenvironment. As stated by Broekman et al. [81], the understanding of how GBMs recruits normal cells in its surroundings to promote growth, sustenance and invasion of the tumor in the brain, as well as the various types of communication and directives exchanged between the tumor surrounding cells, is still the source of many studies In addition, understanding how new compounds, including those of secondary plant metabolism, act to modulate this microenvironment and GBM cells may shed light on new anti-glioma therapies as the ascendent phytotherapy.

## 4. Anti-Glioma Effects of Flavonoids and Their Derivates

**Figure 3:**
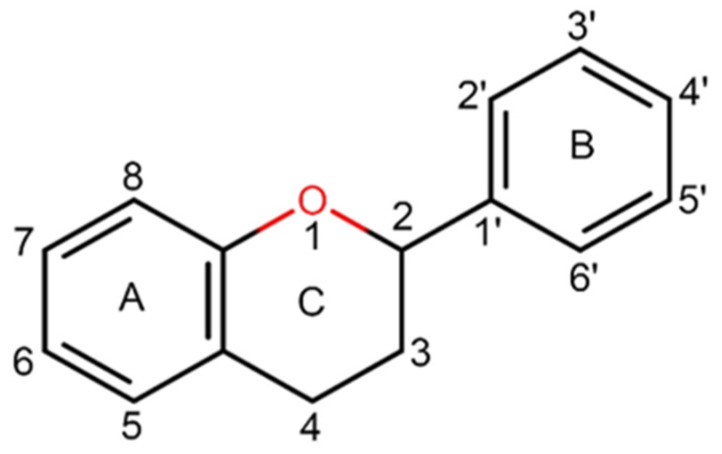
Three-ring structure of flavonoids. (A) The A ring is biosynthesized by the condensation of three moles of malonyl-coenzyme A (CoA) derived from the metabolism of glucose. The C and B rings were also derived from glucose metabolism via the shikimic acid pathway to yield cinnamic acid and its reduced product, coumaric acid (adapted from Formica & Regelson, 1995), (PubChem). An example of flavonoid is presented for each group.

Flavonoids are a group of phenolic secondary metabolites present in several types of plants; They consist of a three-ring structure: two benzene rings (A and B rings) interconnected by a heterocyclic ring (C-ring) (Figure 3) [82]. Flavonoids can be subdivided into several subgroups based on the modifications of their rings [83]. These natural compounds are present in the food of human eating habits mainly in the form of glycosides, but they can also be found in the form of aglycone [84,85]. The aglycone form can be absorbed more easily in the intestine, but most flavonoids are naturally found in [86]. Therefore, some studies suggest that their glycosidic form needs to be hydrolyzed to produce effects in human body [87].

According to structural arrangements of flavonoids, they are divided into seven classes: flavans, isoflavones, flavonols, flavones, flavanones and chalcones [88]. Structural variations that give rise to the diversity of classes result from changes in the hydroxylation pattern and oxidation state of the heterocyclic chain of the pyran [88,89].

Flavonoids play a role in regulating gene expression and metabolism, normally presenting low toxicity due to their low solubility in water and the rapid catabolism of the pyrone nucleus in the liver [90]. Different biological activities have been attributed to flavonoids such as antioxidant and anti-inflammatory, as demonstrated by the flavonols fisetin, kaempferol, morin, myricetin, and quercetin [91], reduced susceptibility to cancer by flavonoids and isoflavonoids ingestion [92], reduced risk of death from coronary and cardiovascular disease attributed to the ingestion of flavanones and anthocyanidins [93].

Flavonoids cross the blood-brain barrier (BBB) [94] and have attracted scientific attention due to their pharmacological properties of acting as anti-glioma agents. Studies demonstrated that flavonoids act on glioma cells, including human GBM cells, affecting countless signaling pathways, including suppressing the activity of phosphatidylinositol 3-kinase (PI3K) [95], inhibiting the expression of extracellular regulatory matrix proteins and metalloproteinases [5], or activating Wnt/β-catenin signaling [96]. Studies describe the antitumor properties of flavonoids, such as jaceosidine [97], hispidulin [98], luteolin [99] and silibinin [100], with antiproliferative, cytotoxic, and cytostatic activities for the ethyl acetate fraction obtained from *Grazielia gaudichaudeana* [101], as shown for in vitro and in vivo models.

### 4.1. Flavans

The biological properties of flavonoids are very different between one flavonoid and another. Flavans are the simplest member of the class. They are derived from benzopyrans with the 2-phenylchroman structural unit type C6-C3-C6. This chemical constituent has varied biological activities and a high degree of structural diversity [102]. The study developed by Maués et al. [103] investigated the properties of flavonoids from the flavan and chalcone classes in C6 rat glioma cells. The prenylated flavan BAS-4, a brosimine B, was extracted from the bark of the *Brosimum acutifolium sub. acutifolium (H.)* and showed evidence of induction of cell death by apoptosis in C6 cells, with loss of mitochondrial integrity, morphological changes such as retraction of the cell body, reduced migration and cell proliferation in a dose-dependent manner. BAS-4 additionally reduced the expression of the protein kinase B/Akt signaling pathway, which is activated in glioma cells, demonstrating its effectiveness with low cytotoxic activity in non-neoplastic cells and a low hemolytic index in red blood cells.

### 4.2. Isoflavones

Isoflavones are analogous to phytoestrogens, with an oxygen atom in the C4 position and the OH radical in the C7 position. These compounds are present mainly in leguminous plants [104]. In this study, biochanin A is the compound that represents the class. According to a study developed by Křížová et al. [105], isoflavones are considered as chemoprotective and can be used as an alternative therapy for a wide range of hormonal disorders, including several cancer types, namely breast cancer and prostate cancer, and other diseases.

### 4.3. Flavonols

Flavonols are the flavonoids most found in food. T heir structure contains a double bond between the C2 and C3 atoms, in addition to the OH radical at the C3 position. As examples, galangin, myricetin, fisetin, icariside II, kaempferol, rutin, 3-O-metil-quercetin and quercetin can be cited, the latter being the compound that leads this class in terms of representation [86]. Galangin, a flavonol of the plant known as Galanga (*Alpinia officinarum*), has shown inhibitory on A-172 human GBM cell proliferation and cell migration in non-toxic concentrations [104]. In addition, the compound induced apoptosis, pyroptosis and autophagy processes in U-251 and U-87 GBM cells [106]. Myricetin also promoted apoptosis in GBM cells, mediated by TNF-related apoptosis-inducing ligand (TRAIL) through the ectopic overexpression of the short cellular FLICE-inhibitory protein (c-FLIP) isoform, in a post-transcriptional and B-cell lymphoma protein 2 (BCL-2) mannercompared tumor but not in healthy human astrocyte cells [107]. Studies show that the regulation of autophagic processes can optimize the cytotoxic effects of antitumor drugs [108]. Other investigations suggest that aberrant changes in the Ras/Raf/MEK/ERK signaling cascade induce and maintain the formation of malignant gliomas [109,110] and may be considered as target signaling pathways of flavonols. The flavonol quercetin promoted cell death through the activation of the mitochondrial pathway in U-373MG cells, with increased expression of p53, which translocated to the mitochondria and simultaneously led to the release of cytochrome c from mitochondria to the cytosol [111]. Bi and collaborators [112] proved the involvement of quercetin in autophagic processes in U-87MG and U-251MG GBM cells. The authors knocked down Beclin 1 gene expression in both strains and used 3-methyladenine (3-MA) and chloroquine (CQ) to inhibit the initial and final stages of autophagy, respectively. They concluded that the inhibitors promoted a dose-dependent increase in the cytotoxicity of quercetin inducing autophagy and apoptosis of glioma cells at a late stage. Quercetin in a dose-dependent manner reduced the levels of proteins p-AKT, p-ERK, BCL-2, MMP-9 and fibronectin (FN), suppressing the pathways of RAS/MAPK/ERK and PI3K/AKT signaling [113] and the expression of phospholipase D1 (PLD1) induced by NF-kB, inhibiting the activation and invasion of MMP-2 [114]. In addition, quercetin also sensitized U-87MG, U-251MG and A-172 glioma cells to the apoptosis mediated by TRAIL, but not in U-373GM cells, by survivin suppression [107] and anti-apoptotic protein [115]. Treatment with the flavonol kaempferol promoted TRAIL-mediated apoptosis in U-251MG and U-87MG cells by proteasomal degradation of survivin; however this effect was not observed in U-373 cells [107]. Some flavonols, such as fisetin (3,3′,4′,7-tetrahydroxyflavone), act by phosphorylating extracellular signal-regulated kinases (ERK1/2), also promoting the inhibition of a disintegrin and the metalloproteinase 9 (ADAM9) [5,115], a protein associated with the GBM invasion and the degree of malignance, acting as a factor of prognosis in gliomas [116]. Fisetin also evoked the reduction of migration of human GL-15 cells associated with the decreased 8401 GBM cell proliferation and invasion at non-toxic concentrations [117]. Rutin (quercetin-3-O-rutinoside), abundant in beans of the Brazilian plant *Dimorhandra mollis Bent.*, presented antiproliferative, proapoptotic and morphogenic effects on GL-15 human GBM cells, associated with decreased levels of ERK1/2 phosphorylation (P-ERK1/2) and accumulation of cells in the G2 phase of the cell cycle [118]. Rutin also promoted reduction of GBM cell viability by inhibiting cell metabolism and migration, related to a reduction in filopodia-like structures on the cell surface, reduction of metalloproteinase (MMP-2) expression and activity, as well as an increase in intra- and extracellular expression of fibronectin, and intracellular expression of laminin, both matrix proteins [5]. Antitumor properties of the flavonols rutin and quercetin were also investigated in C6 GBM cell monoculture and co-culture of C6 cells. It was observed that both flavonoids induced inhibition of tumor cell proliferation and migration [10]. These same authors followed the formation of tumors in the brain of Wistar rats, after 30 days of U-251 GBM cell xenotransplantation, under control conditions or pretreated for 24 h, with rutin or quercetin, and thus observed a reduction of tumorigenesis in the microenvironment of implantation of tumor cells for those xenotransplanted animals with cells treated by flavonoids [10].

Icariside II (ICA II) is a bioactive flavonol derived from *Epimedium koreanum* that is used in Traditional Chinese Medicine. The study by Quan and collaborators [119] investigated the mechanisms through which this flavonoid induces apoptosis and cell cycle arrest in U-87 and A-172 GBM cells. They found that the effects depended on inhibiting phosphorylation and activating Akt signaling pathway, inducing the expression of apoptosis-related proteins such as cleaved caspase-3, cytochrome c, poly ADP ribose polymerase (PARP) and p53, in addition to promoting Forkhead Box O3A (FOXO3) to be transported from the cytosol to the nucleus leading to the transition of p21 and p27 proteins.

The class of flavanols, also called monomeric catechins (flavan-3-ols), has several substituents. The hydroxyl group is always linked to position 3 of ring C and there is no double bond between positions 2 and 3 [83]. They are abundant in their polymerized forms such as oligomers in natural food products, in juices of fruit [120], cocoa and tea [121]. Green tea has phenolic compounds like epigallocatechin-3-gallate (EGCG) [122]. This flavonoid induced rapid apoptosis in glioma cells resistant to TRAIL, in addition to the downregulation of phosphoprotein enriched in astrocytes 15 (PEA15), as key regulators of cell death [108].

Flavonolignans are composed of a flavonoid unit (taxifoline) and a phenylpropanoid unit (a portion of coniferyl alcohol), linked by an oxeran ring [123]. Milk thistle (*Sylibum marianum*) and its seeds contain the entire family of natural flavonolignan compounds. Silibinin is a flavonolignan found in *S. marianum* [124]. This flavonoid upregulated the death receptor 5 (DR5) related to TRAIL inducing C/EBP Homologous Protein (CHOP) transcription factor-dependent apoptosis in U-251MG; U-87MG, A-172, and U251N GBM cells, but not in healthy human astrocyte cells, in addition to reducing the expression of the proteasome-degraded anti-apoptotic FLICE-inhibitory protein long-form (FLIPL), FLICE-inhibitory protein short form (FLIPS) and survivin proteins [125]. Another mechanism of action of silibinin is the induction of caspase-dependent cell death via Ca^2+^ signaling mediated by ROS/mitogen-activated protein kinase (MAPK), as demonstrated in vitro. In this study, the induction of cell death by silibin was effectively prevented by the calpain inhibitor [126]. Recently, the induction of U-87MG GBM cell death was shown to depend on calpain activation, activating the mitochondrial apoptotic pathway, nuclear translocation of the apoptosis-inducing factor (AIF), which mediated by the activation of PKC and the generation of ROS, as well as by PI3K and Fox M1 inactivation [127,128].

### 4.4. Flavones

Flavones are the most common flavonoids present in effective concentrations in foods. Flavones present a double bond between C2 and C3 atoms, in addition to a carbonyl group present in the C4 position. This class consists of jaceosidine, hispidulin, linarin, nobiletin, wogonin, oroxylin A, wogonoside, hydroxygenkwanin and lycoflavone C, as well as apigenin and luteolin, which the most studied compounds of this class [86,88]. The methoxylated flavones casticin (5,3′-dihydroxy-3,6,7,4′-tetramethoxyflavone) and penduletin (5,4′-dihydroxy-3,6,7-trimethoxyflavone), isolated from *Croton betulaster Müll Arg.*, a plant utilized in popular medicine in Brazil, inhibited the metabolic activity and, in a dose-dependent manner, the proliferation of GL-15 GBM cells, driving cells to apoptosis [6]. Moreover, linarin (acacetin-7-O-rutinoside), isolated from *Buddleja officinalis*, suppressed cell proliferation and migration of A-172, U-251 and H4 GBM cells. Linarin promoted tumor reduction by r p65 and p53 expression regulation and inducing apoptosis in a dose-dependent action. Further, treatment with linarin resulted in TRAIL-induced apoptosis in vitro, regulated by the generation of reactive oxygen species (ROS) [70,129]. Nobiletin (3′,4′,5,6,7,8-Hexamethoxyflavone), also a polymethoxylated flavone, is present in the human diet, such as citrus fruits [130]. It exerts several pharmacological activities, including action in brain tissue [131,132]. Aoki et al. [133] demonstrated that nobiletin promoted inhibitory effects on Ras activation, suppression of cell proliferation, reduction of MEK, Akt and ERK phosphorylation levels, through Ca^2+^ influx and Ca^2+^ sensitive protein kinase C (PKC) activation. Thus, modulation of the Ras/Raf/MEK/ERK signaling cascade by flavonoids may be a strategy in the control of proliferation of gliomas, [133] considering that flavone wogonin reduced cell viability and facilitated cell death in two strains of GBM (U-251 and U-87), but not in primary human astrocytes, inducing the activation of pro-caspase-9, caspase-3 and PARP mediated by ROS generation. Moreover, it was also demonstrated that wogonin induced a reduction of cell proliferation of F-98 GBM cells, associated with the inhibition of the AKT pathway [134]. Its glycosides such as flavone wogonoside induced death in SHG-44, A-172, U-87 MG and U-251 MG GBM cells, the last being more sensitive to treatment, cells presenting features of autophagy followed by mitochondrial apoptosis associated with the activation of the p38 MAPK and inhibition of PI3K/Akt/mTOR/p70S6K pathways [134]. The flavone oroxylin A inhibited the growth of U-251, U-118, U-87 GBM cells and promoted cell death by autophagy involving Beclin 1, and by blocking AKT and ERK activation and phosphorylation of the mTOR-STAT3-Notch-1 pathway [135].

Apigenin (5,7,4′-trihydroxyflavone), extracted from *Croton betulaster Mull*, was found to have the ability to reduce the viability and proliferation of C6 glioma cells in a time- and dose-dependent manner, inducing cell accumulation in the G0/G1 phase of the cell cycle and inhibiting glioma cell migration efficiently [136]. During a study developed by Coelho et al. [6], it was possible to verify the antiproliferative effect of the flavone apigenin on C6 cells and the anti-migratory effect through a conditioned medium of microglia cells treated with apigenin. The anti-glioma effect of apigenin can be enhanced by making concomitant use of another flavonoid. The study developed by Wang et al. [137] investigated the antitumor effect of the flavone hydroxygenkwanin (HGK) isolated and administered together with apigenin. HGK shows the antiproliferative activity of C6 cells through activations induced by the tumor necrosis factor-alpha (TNF-α) of caspase 3/8, which results in apoptosis. However, when apigenin was administered simultaneously with HGK there was a synergistic effect with a decrease in cell viability in a dose-dependent manner, loss of mitochondrial membrane potential, stopping the S phase of the cell cycle and inducing positive regulation of the level of TNF-α, activation of caspase 3/8 and negative expression of the BCL-XL protein.

When compared to apigenin with prenylated flavonoids such as licoflavone C (8-prenylapigenin) and isobavachin (8-prenylliquiritigenin), a flavone and a flavanone, respectively, in C6 cells, one can observe that the prenylated flavonoid shows a significant reduction in cell viability and cytotoxicity mediated by the induction of apoptotic cell death, increasing the activity of caspase 3/7. These effects appear to be derived from C8 prenylation, which increases the hydrophobicity and consequently the toxicity of flavonoids such as apigenin and liquiritigenin [138]. A synthesis of studies that demonstrated effects of flavonoids in glioma models can be seen in Table 1.

Quercetin, rutin, luteolin and apigenin, two flavonols and two flavones respectively, are abundant flavonoids in the human diet [139] and can be present in food sources such as the leaves of parsley, chamomile, green tea, black tea, citrus fruits, red and white wine, different types of rice, in wheat used to make various dishes and several other common everyday foods [140,141,142,143,144,145,146]. These flavonoids are involved in inhibition of mechanisms of tumor cell survival, proliferation, and death [147], growth inhibition, regulation of pro-angiogenic factors, such as VEGF, and transforming growth factor β (TGF-β) in GBM [148] and not altering the viability of normal glial cells [6,149].

### 4.5. Flavanones

Flavanones have characteristics that make them different from flavonols and flavones, such as the lack of a double bond between the C2 and C3 atoms, lack of substituents in the C3 position, in addition to the presence of a chiral carbon in the C2 position [150]. This class includes the compounds naringenin ((2S)-4′,5,7-trihydroxyflavan-4-one), 8-prenylnaringenin and 8-prenylliquiritigenin. Naringenin and its prenylated derivative 8-prenylnaringenin demonstrated important activity on brain tumors. However, the derivative had greater inhibitory effects on U-118 GBM cells than did the flavonoid itself [151]. The 8-prenylnaringenin derivative also had a 37% higher accumulation in GBM than in normal cells. This potentiation of the antiglioma effect may be related to the prenyl group of the compound that leads to an increase in hydrophobicity, retaining the compound in the intracellular compartment for a longer time and thus increasing its activity. In another study, Aroui and collaborators [152] demonstrated that naringenin inhibited the invasion and migration of U-251 GBM cells through the negative regulation of MMP-2 and matrix metalloproteinase-9 (MMP-9) expression and inactivation of p38 signaling pathway, involved in the regulation of many cellular processes, including differentiation, growth and cell death [153].

### 4.6. Chalcones

Chalcones, precursors of flavonoids, are α, β-unsaturated ketones that have two aromatic rings (A and B). These compounds are open-chain flavonoids and have a diversity of substituents; therefore, they are the most diverse group of flavonoids [154,155]. As an example, we presented in this study the chalcone isoliquiritigenin (ISL). ISL inhibited the proliferation of U-87 GBM cells in a time- and dose-dependent manner, induced apoptosis, blocked the progression of the cell cycle in phases S and G2/M [156]. ISL also induces a reduction in diameter and number of tumorspheres formed from the GSCs, presenting a differentiation-inducing effect in human GSCs, with high levels of protein expression of differentiation markers and the Notch signaling pathway downregulation [157].

The mixture of different compounds can cause synergism, reduction or inhibitory effects of the active substances [158]. *Sideritis scardica* (mountain tea) is an endemic plant in southeastern Europe used both medicinally and as a beverage. A *S. scardica* crude ethanol extract was partitioned, and the authors demonstrated that the cytotoxic effects against C6 rat glioma cells attributed to luteolin, luteolin-7-O-glycoside and apigenin. The most cytotoxic extracts against C6 GBM cells were diethyl ether and ethyl acetate, with the most significant effect inducing ROS and autophagy and reducing the tumor cell viability [159]. Another extract, such as from the rice bran Njavara (Shastika Shali), rich in flavonoids, showed an antiproliferative effect in the same GBM cells [160]. Flavonoids such as luteolin, quercetin, 3-O-methyl-quercetin and achyrobichalcone can be found in the medicinal plant *Achyrocline satureioides*. Both the isolated flavonoids, as well as a *A. satureioides* extract containing flavonoids, reduced the proliferation of U-251MG, U-87MG and C6 cells, altered cell morphology and induced activation of the cell death pathway by apoptosis. This extract was also able to reduce one of the main signal transduction pathways for cancer, MYC and MAP kinases (ERK and Jun N-terminal kinases (JNK) [161].

Dell’Albani et al. [162] proposed that brome and/or hydrophobic acyl groups in specific positions of a flavonoid molecule with antiglioma effect could provide greater anti-tumor capacity. The authors modified the structure of quercetin with the addition of bromine, demonstrating that there were dose-dependent effects, such as reduced viability and apoptosis, in addition to more significant morphological changes in cells when compared to the effects of the original flavonoid. The derivatives 3-O-decanoylquercetin and 6,8-dibromo-3-O-palmitoylquercetin with more significant activity also affected cell survival, differentiation and migration signaling pathways, reducing ERK and AKT phosphorylation, in addition to the activation of caspase-3, indicating apoptosis.

Natural products that include flavonoids in their chemical composition are propolis and bee honey. Pinocembrin, crisin, galangin, alpinetin, dillenetin and isorhamnetin flavonoids were identified in a sample of propolis ethanolic extract (EEP) collected in Mexico, while phytochemicals galangin, ferulic acid, syringic acid, caffeic acid had cytotoxic action in several cancer lines. The results showed that EEP restricts the proliferation of C6 glioma cells in vitro as efficiently as temozolomide does, revealing concentration-dependent cytotoxicity [163]. Stingless bee honey, on the other hand, has its antitumor activity attributed to the antioxidant capacity of the phenolic and flavonoid compounds present. The data in this study reveal that honey has a cytotoxic and proliferation-inhibiting effect on U-87 GMB cells in a time- and dose-dependent manner, in addition to apoptosis-induction [164].

Several flavonoids are found in the plant extracts of the genus *Scutellaria*. The bioactive flavones in *Radix Scutellariae*, the dried root of *S. baicalensis Georgi*, are found in the form of aglycone (baicalein, wogonin, oroxylin A) and glycoside (baicalin, wogonoside or oroxylin A-7-glucuronide), wogonin being the main component, while the presence of apigenin and luteolin has also been described [91,165,166,167]. Some studies have investigated the activities of these aglycones and glisosides. The study by Parajuli et al. [167] demonstrated the anti-glioma activity of a leaf extract from the *S. ocmulgee* (SocL). The extract promoted the inhibition of tumor growth in vivo (F-98 cells injected by stereotaxis), in addition to increasing the survival in rats. In vitro, the extract inhibited proliferation of F-98 GBM cells in a dose-dependent manner, associated with the inhibition of AKT, glycogen synthase kinase 3 (GSK-3) phosphorylation and NF-κB.

## 5. Current Treatment of GBMs and Association with Natural Products

At present, treatment of GBMs involves surgery, radiation and chemotherapy. Maximal resection of the tumor area ensures reduced intracranial pressure and longer patient survival when compared to incomplete resection [168]. The surgery is followed by radiotherapy using 2 Gy per fraction per day, for 5 days/week for 6 weeks, with a total dose of 60 Gy together with chemotherapy, simultaneously [169,170]. The gold standard treatment for GBM in newly diagnosed patients is the use of Temozolomide (TMZ), at a dose of 75 mg/m^2^, based on the body surface, daily, for six weeks with focal radiotherapy, as recommended by the Food and Drug Administration of the United States of America (FDA). Adjuvant treatment consists of a 4-week rest period after simultaneous radiation therapy, with an increase in dose to 150 mg/m^2^ daily for five days in a 28-day cycle. After this period, 150–200 mg/m^2^ daily are administered for 5 days over 28 days, consisting of a new cycle [169].

TMZ is a DNA alkylating agent with nonspecific actions on the cell cycle, which was approved in 2000 as a therapeutic agent for the treatment of high-grade malignant gliomas [171]. Due to its lipophilic nature [172], this drug has good oral bioavailability and penetration into the BBB [173], but it causes adverse effects such as hematological toxicity, gastrointestinal problems and nervous system disorders [170]. However, the poor prognosis of GBM and resistance to treatment requires novel therapeutic strategies and preventive targets, once that those are more effective and less toxic.

The association of TMZ with natural compounds, including flavonoids and their derivatives, has been considered in several GBM cells models in vitro. Studies have shown that combinations can reduce the viability of TMZ-resistant cells, such as the co-treatment of U-87 GBM cells with isoflavone biochanin A [172]. A study by Souza et al. [161] demonstrated that the co-treatment with TMZ associated with the extract from Achyrocline satureioides, and its main flavonoids luteolin, quercetin, 3-O-methyl-quercetin and achyrobichalcon, led to prolonged inhibition of GMB cell growth and an increase in the chemotherapeutic effect, without affecting the viability of other cells of the CNS [161]. The study by Wang and collaborators [174] revealed that the therapy with flavonoid ISL concomitant with TMZ significantly increased the survival of rats when compared to monotherapy, also increasing the concentration of TMZ in glioma tissues, inhibiting tumor growth and inhibiting several mediators such as COX-2, mPGES-1 and CYP4A, which decreases the production of FGF-2, TGF-β and VEGF via Akt signaling.

Like TMZ, flavonoids also have a synergistic activity with other chemotherapy drugs. The flavonol kaempferol potentiated the toxic effect of doxorubicin, promoting apoptosis [175], and silybin when combined with arsenic trioxide (ATO), also promoting apoptosis, decreasing the level of expression of MMP 2 and 9, survivin and BCL-2 [176]. These findings put in evidence that flavonoids are promising for the treatment of GBMs and other glial tumors, and more studies are necessary to explore the molecular mechanisms involved in their antiproliferative effect in vitro as well as their efficacy in vivo in association with the current therapy.

## 6. Neuroimmunomodulatory Action of Flavonoids and Derivatives

GBMs are tumors that have a high ability to migrate, invade and proliferate in the healthy tissue, which greatly impairs their treatment [169,171]. These characteristics are associated with the surrounding proinflammatory microenvironment, which contains growth factors and pro-inflammatory mediators. These molecules are expressed and released by tumor cells, but local immune cells can react and secrete mediators in the tumor environment. Pro-inflammatory cytokines, growth factors, nitric oxide (NO), ROS, hydrolytic enzymes and MMPs are examples of molecules synthesized and secreted by defense cells and tumor cells, which contribute to the biochemical composition and inflammatory characteristic of the tumor microenvironment [177]. Gliomas release growth factors and overexpress their receptors, generating paracrine and autocrine signaling [178]. Faced with tumor hypoxia, VEGF is released and mediates the growth of new vessels. Studies have demonstrated that VEGF has a key role in the tumor growth process through angiogenesis [179]. TGF-β is predominantly immunosuppressive, abundantly released by glioma and surrounding-microglia. It regulates the undifferentiated state of glioma stem cells, contributes to angiogenesis, tumor growth and invasion. The tumor cell mobility is increased by the regulation of surface integrins that maintain contact with the extracellular matrix (ECM) scaffold [180]. Studies conducted with in vitro and in vivo models of GBM have also shown the antitumor potential and immunomodulatory activity of flavonoids and their derivatives. Naringenin, for example, was able to inhibit the migration and invasion of 8901GBM cells, reducing the expression of the metalloproteinases MMP-2 and MMP-9, as well as the activity of ERK and p38 [181]. Among nine substances that reduced VEGF secretion in U343 and U118 GBM cells, the flavonoids naringin and rutin were more efficient, being effective in lower concentrations [182]. In U87 and Hs683 GBM cells, nobiletin inhibited cell migration and proliferation, interrupting the cell cycle in the G0/G1 phase. It also prevented phosphorylation, that is, the activation of p38, ERK and JNK, and reduced the expression of cyclin-dependent kinases 1, 2 and 4, as well as the E2 promoter binding factor (E2F1), which promotes cell proliferation [183].

Several non-neoplastic cell types also comprise the GMB microenvironment, e.g., fibroblasts, endothelial cells and immune cells. Microglia and macrophages account for 30–50% of glioma cells and, therefore, greatly determine tumor microenvironment and development. This influence makes these cells important targets for the development of therapeutic interventions in association with therapies directed to neoplastic cells [184]. Microglia are motile cells with a compact body and ramified projections, which actively monitor/survey the environment for cues regarding the health state/status of the CNS. This surveillance is enabled/promoted/accomplished by a variety of surface receptors, which integrate environmental cues on the health status of surrounding cells. In response to signals that indicate homeostasis disturbance, microglia (as well as astrocytes) change the morphology, physiology and gene expression defined as glial activation or reactivity. These changes allow glial cells to perform functions (e.g., phagocytosis, the release of cytokines and chemokines, antigen presentation to T cells) that aim to promote clearance of injured cells and damage restraint [44]. Although microglial subpopulations in activated or surveillance states differ according to brain regions or activation stimuli [185], they have predominantly pro- or anti-inflammatory properties, which are termed M1 and M2 microglia, respectively [184]. However, this paradigm among these different phenotypes presented has been of great interest in studies of neurodegenerative diseases [186]. It has already been shown that GBMs can induce polarization of tumor-infiltrated microglia/macrophages to the M2 profile in an in vivo model, what favors tumor growth [187]. Meanwhile, in experiments performed with microglia from rats, it was found that these cells, when treated with the conditioned medium that mimic an early stage of the disease, tended to polarize to the M2 profile. However, when the microglia were treated with medium obtained after the stimuli with LPS, they presented both M1 and M2 phenotypes [188]. These findings put in perspective the modulation of inflammatory profile of microglia as another strategy to control GBM.

In the tumor microenvironment, in addition to these factors, the interleukin 6 (IL-6) is an important pro-inflammatory cytokine, involved in the response of T helper 2 (Th2) cells. In healthy nervous tissue, it is involved in astrogliosis reactions, in the activation of the inflammatory response to pathological damage [189]. Meanwhile, in CNS pathologies, as in gliomas, this cytokine is positively regulated, contributing to the activation of the signal transducer and activator of the transcription 3 (STAT3) signaling pathway. The STAT3 cascade is associated with the escape of the tumor from immune surveillance, and demonstrating an important role in inducing cell proliferation, migration and invasion tumors, as well as promoting angiogenesis and resistance to apoptosis [91,190,191,192,193,194].

Jang et al. [195] showed that luteolin (3′,4′,5′,7′- tetrahydroxyflavone), a flavonoid found in green pepper, parsley and chamomile tea inhibited the production of IL- 6 in primary cultures of microglia from mice and cultures of human microglial cells BV-2, after being stimulated with lipopolysaccharide (LPS). The authors observed that, in addition to reducing cytokine levels, there was also a reduction in messenger RNA (mRNA) expression for IL-6, by inhibiting the signaling mechanism of the JNK pathway and activating the activator protein 1 (AP-1). In another study, it was shown that quercetin inhibited the IL-6-induced STAT3 signaling pathway in T98G and U87 GBM cells, markedly reducing proliferative and migratory properties of GBM cells [192].

Interleukin 1 Beta (IL-1β), a pro-inflammatory cytokine, has an important role in triggering several oncogenic processes in the tumor microenvironment, such as secretion of cyclooxygenases (COXs) and increase in hypoxia-inducible factor 1α (HIF-1α) activity [196,197]. This cytokine is important for the activation of some signaling pathways such as NF-κB (which promotes the production and release of pro-inflammatory mediators and, therefore, is intensely involved in tumor development and progression) and MAPK (which collaborates for tumoral invasion [198,199,200]. Furthermore, IL-1β contributes to the development of a microenvironment that is favorable to glioma migration and invasion [201].

TNF-α is a pro-inflammatory cytokine with diverse roles, e.g., it can favor tumor development, angiogenesis, as well as apoptosis and recruitment of macrophages (resident of the CNS or infiltrates). The variety of effects of TNF-α results from its ability to interact with a number of receptors, leading to the activation of several intracellular pathways [202]. Some authors address the role of flavonoids as naringin, apigenin, fisentin and quercetin in the regulation of IL-1β in the tumoral context [203,204]. In addition, it was shown that luteolin can inhibit the IL-1β-mediated phosphorylation of κβ inhibitor in U-87 GBM cells [205]. Besides that, rutin has stood out as a potent immunomodulatory agent. It was demonstrated that rutin can reduce the mRNA levels of IL-1β in C6 lineage cells. This same effect was shown about the IL-6 expression and TNF-α [10]. Inflammation-related mediators released by tumor cells inhibit T helper 1 (Th1) cells, e.g., IL-2, Interferon-gamma (INF-γ) and TNF-α, and induce Th2 peripheral cytokines, e.g., IL-10, IL-6 and IL-4. In response to acute inflammation, naive T cells differentiate into immunocompetent cells [206]. The expression of TNF-α and IL-6 is promoted by the activation (phosphorylation) of p38 MAPK, which is translocated to the nucleus and activates transcription factors, such as NF-κβ. Transcriptional changes resulting from the activation of MAP p38 result in the expression of pro-inflammatory cytokines and in the synthesis of ECM components, such as fibronectin, which was reduced by apigenin [207]. The treatment of cells of the C6 lineage with apigenin increased the production and release, by these cells, of TNF-α [137]. Apigenin treatment of microglia/C6 co-cultures also induced preferentially a reduction in the viability of C6 cells and increased microglia-activated phenotype, associated with a change in the balance of TNF/IL-10 levels, demonstrating that the flavonoid restores the immune profile of microglia against glioma cells [6]. The TNF receptor can trigger either the production of pro-inflammatory mediators, e.g., through the activation of NF-κB, or the extrinsic pathway of apoptosis. Certain ligands of the TNF superfamily can lead to the activation of the extrinsic pathway of apoptosis, such as TNF-α, Fas (CD95L), or TRAIL [208].

Another important cytokine in this microenvironment is the IL-8 secreted by tumor cells. It is also known as a chemokine involved in tumorigenic and pro-angiogenic processes. Besides that, it is an important leukocyte chemotactic factor [209,210,211]. About IL-8 regulation, it was found that miRNA-93 plays a crucial role in the process of reducing its release in human glioma cells of the U251 and T98G lineages [212]. However, some researches report that flavonoids, such as luteolin, can be involved in this tumoral process, regulating the IL-8 release as well [205]. It was possible to verify a decrease in the IL-8 levels in C6 cells culture when subject to the co-treatment with flavonoid quercetin and AG490, a Janus kinase 2 protein (JAK2) specific inhibitor [137].

IL-10 is a cytokine with anti-inflammatory properties, which promotes cell repair after a cytotoxic, pro-inflammatory response. Molecular mechanisms involving this cytokine have been increasingly elucidated due to a great interest in understanding its involvement with several events, to envision new immunological intervention strategies [213,214]. Among its many functions is the participation in activating the signaling pathway as STAT3, resulting in negative regulation of macrophage activation [215]. A study conducted by da Silva et al. [10] showed that flavonoid rutin induces a microglial polarization to the M2 profile when these cells were stimulated with LPS. In addition, it reduces the levels of secretion of IL-6, IL-1β and TNF (inflammatory cytokines) and is also capable of increasing the production of the regulatory cytokine IL-10. Moreover, in a more recent study developed by da Silva et al. [10], it was demonstrated that the treatment of rat C6 glioma cells with rutin or with its aglycone quercetin induced the inhibition of proliferation and migration, and also induced microglia chemotaxis that was associated to the upregulation of TNF and the downregulation of IL-10, at protein and mRNA expression levels, associated with the upregulation of mRNA expression for chemokines CCL2, CCL5 and CX3CL1. The authors also demonstrated the immunomodulatory effects of the treatment of human U251 and TG1 glioblastoma cells with both flavoinds, negatively modulating the expression of mRNA for IL-6 and IL-10 and positively the expression of mRNA for TNF. Treatment of microglia and C6 cells with rutin or quercetin either in co-cultures or during indirect interactions via conditioned media, reduced proliferation and migration of glioma cells, and directed microglia towards an inflammatory profile, characterized by increased expression of mRNA for IL-1β, IL-6, IL-18, and decreased expression of mRNA for nitric oxide synthase 2 (NOS2) and prostaglandin-endoperoxide synthase 2 (PTGS2), arginase and transforming growth factor beta (TGF-β). This study also demonstrated that the treatment of U251 cells with flavonoids rutin or quercetin also reduced tumorigenesis when the cells were xenotransplanted in rat brains, also directing microglia and astrocytes to an inflammatory profile in the microenvironment of tumor cell implantation as well as in the brain parenchyma not favorable to the glioma growth.

**Figure 4:**
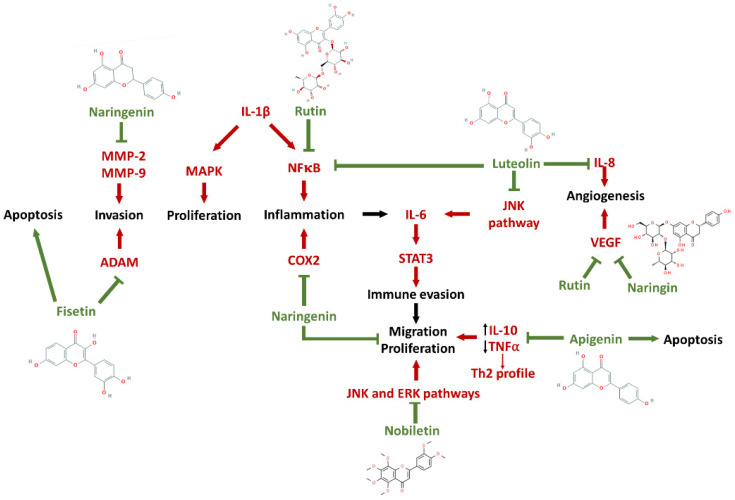
A schematic representation of the possible mechanisms of action of selected flavonoids. Note that these flavonoids act in different signaling pathways, reducing inflammation, migration, proliferation, invasion, angiogenesis and inducing apoptosis in tumor cells.

These data suggest the immunomodulatory properties of several flavonoids on the production and secretion of cytokines, summarized in Table 2. Flavonoids can make the tumoral microenvironment less favorable to the expansion of GBM cells, also reducing its ability to interact with cells present in its tumor niche. Future studies will further elucidate the effects of flavonoids in the GBM context, in terms of their immunomodulatory activity. However, the antitumoral and wide beneficial effects of flavonoids have long been demonstrated in several biological experimental settings. It has been demonstrated that flavonoids target multiple pathways, both to inhibit glioma development and survival, as well as to revert immunosuppression in immune cells in the glioma microenvironment. This poses flavonoids as strong candidates for the development of more efficient anticancer drugs. A schematic overview of immunomodulatory actions of flavonoids on glioma cells can be seen in Figure 4.

## 7. Conclusions

Current findings show that flavonoids present potential therapeutic aplications as antigliomatic drugs. The cytotoxicity for glioblastoma cells has been pointed as a strategy for bioprospection. Recent studies, as discussed here, evidenced the modulation of microglia response as a toll to provide an antitumor reprogramming microenvironment, which in association with current therapy can be a promising strategy against drug resistance. However, the biology of microglia interaction with GBM, and involving other cell population in the tumor microenvironment, must be considered for the research on flavonoid application as adjuvant therapy.

## Figures and Tables

**Table 1:** A synthesis of studies that demonstrated effects of flavonoids in glioma models.

Flavonoid	Origin	Molecular Structure *	Concentration	Glioma Models	Effects
Biochanin A	Sigma-Aldrich (St. Louis, MO, USA)	https://pubchem.ncbi.nlm.nih.gov/compound/Biochanin%20A	Biochanin A + TMZ (70 µM + 70 µM)	U-87 MG	Reduction of cell viability (Desai et al., 2019)
Isoliquiritigenin	Sigma-Aldrich (St. Louis, MO, USA)/ *Glycyrrhiza* spp.	https://pubchem.ncbi.nlm.nih.gov/compound/Isoliquiritigenin	10 and 20 mg/kg 0–80 µmol/L 10 to 160 µM	C6 U-87 SHG-44 GSCs	Antiangiogenic, antiproliferative, induction of apoptosis, stimulation of cell differentiation synergistic activity with TMZ (Zhou, Song & Yang, 2013; Lin et al., 2018, Wang et al., 2019)
Kaempferol	Sigma-Aldrich (St. Louis, MO, USA)	https://pubchem.ncbi.nlm.nih.gov/compound/Kaempferol	50 to 200 μmol/L Kaempferol + Doxorubicin (50 µmol/L + 1 µmol/L)	U-87 MG U-87 U-251	Induction of apoptosis (Sharma et al., 2007; Siegelin et al., 2008)
Silibinin	Sigma-Aldrich (St. Louis, MO, USA)	https://pubchem.ncbi.nlm.nih.gov/compound/Silibinin	200 mg/kg/day 150 μmol/L 30 to 200 μM Silibinin + ATO (75 μM + 1 or 2 μM)	U-87 MG T98-G U-251 MG A-172 U251N U-251	Apoptosis, reduction of the growth of tumor, downregulation of antiapoptotic proteins (Son et al., 2007; Kim et al., 2009; Jeong et al., 2011; Dizaji et al., 2012; Zhang et al., 2015; Chakrabarti, Mrinmay & Swapan, 2016)
Jaceosidine	Leaves of *Artemisia argyi*	https://pubchem.ncbi.nlm.nih.gov/compound/Jaceosidine	100 * * μM/L	U-87	Apoptosis (Khan et al., 2012)
Hispidulin	Tocris Bioscience (Bristol, U.K.)	https://pubchem.ncbi.nlm.nih.gov/compound/Hispidulin	60 and 40 μM	GBM 8401 GBM 8901	Antiproliferative activity, suppression of mTOR signaling, growth arrest and apoptosis (Lin et al., 2010)
Galangin	Sigma-Aldrich (St. Louis, MO, USA)	https://pubchem.ncbi.nlm.nih.gov/compound/Galangin	5 to 300 μM	A-172 U-251 U-87 MG	Induction of apoptosis, pyroptosis and autophagy, inhibition of cell migration (Lei et al., 2018; Kong et al., 2019)
Linarin	Chengdu MUST Biotechnology (Chengdu, China)	https://pubchem.ncbi.nlm.nih.gov/compound/Linarin	5, 80 and 100 μM 12.5, 25 and 50 mg/kg/gavage	A-172 U-251 U-87 MG	Apoptosis, decrease in tumor growth, suppression of cell proliferation and migration (Zhen et al., 2017; Xu et al., 2017)
Myricetin	Sigma-Aldrich (St. Louis, MO, USA)	https://pubchem.ncbi.nlm.nih.gov/compound/Myricetin	50 to 200 μM	U-251 LN-229	Apoptosis mediated by TRAIL, suppression of c-FLIP (Siegelin et al., 2009)
Fisetin	Sigma-Aldrich (St. Louis, MO, USA)	https://pubchem.ncbi.nlm.nih.gov/compound/Fisetin	10 to 40 μM	GBM 8401	Reduction of cell migration and invasion (Chen et al., 2015)
Icariside II	*Epimedium koreanum*	https://pubchem.ncbi.nlm.nih.gov/compound/Icarisid-II	20 or 40 μM	U-87 A-172	Reduction of proliferation and migration cell, apoptosis and cell cycle arrest (Quan et al., 2017)
Nobiletin	*Citrus depressa*	https://pubchem.ncbi.nlm.nih.gov/compound/Nobiletin	10–100 μM	C6	Suppression of RAS (Aoki et al., 2013)
*Scutellaria ocmulgee* SocL (leaf extract)	Cultured at Specialty Plants House	-	100 mg/kg/gavage 15.6 to 500 μg/mL	F98	Inhibition of tumor growth, cell proliferation and phosphorylation of AKT, GSK-3 and NK- κB (Parajuli et al., 2011)
Wogonin	Sigma-Aldrich (St. Louis, MO, USA)	https://pubchem.ncbi.nlm.nih.gov/compound/Wogonin	12.5 to 100 μM	U-251 U-87 F98	Reduction of cell viability; facilitation of cell death, inhibition of cell proliferation an inhibition of the AKT pathway, activation of pro-caspase-9, caspase-3 and PARP (Parajuli et al., 2011; Tsai et al., 2012)
Oroxylin A	*Radix Scutellariae*	https://pubchem.ncbi.nlm.nih.gov/compound/Oroxylin-A	0 to 200 µM	U-251 U-118 U-87	Inhibition of cell growth and AKT and ERK/mTOR-STAT3-Notch-1 (Zou et al., 2015)
Wogonoside	Shanghai Tauto Biotech Co., Ltd. (Shanghai, China)	https://pubchem.ncbi.nlm.nih.gov/compound/Wogonoside	100 to 500 μM	U-251 MG SHG-44 A-172 U-87 MG	Cell death, autophagy (Zhang et al., 2014)
Naringenin	Sigma-Aldrich (St. Louis, MO, USA)	https://pubchem.ncbi.nlm.nih.gov/compound/Naringenin	1 to 500 μM	U-118 MG U-251	Inhibition of cell invasion and migration (Aroui et al., 2016; Stompor, Uram & Podgórski, 2017)
8-Prenylnaringenin	Demethylation of isoxanthohumol	https://pubchem.ncbi.nlm.nih.gov/compound/8-Prenylnaringenin	1 to 500 μM	U-118 MG	Greater inhibitory effect than naringenin (Stompor, Uram & Podgórski, 2017)
(2*S*)-7, 4′-dihydroxy-8-(3″3″-dimethylallyl)-flavan (BAS-4)	*Brosimum* *acutifolium* *sub. acutifolium* (H.) bark	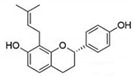	25 to 150 μM	C6	Antiproliferative and inhibitory effects on migration and apoptosis induction (Maués et al., 2019)
Mangiferin	Sigma-Aldrich (St. Louis, MO, USA)	https://pubchem.ncbi.nlm.nih.gov/compound/Mangiferin	50–100 μM	U-87	Apoptosis and inhibition of MPP-9 expression(Xiao et al., 2015)
Epigalocatechin-3-gallate (EGCG)	Sigma-Aldrich (St. Louis, MO, USA)	https://pubchem.ncbi.nlm.nih.gov/compound/Epigallocatechin-gallate	20 μM	U-87 A-172 U-251	Apoptosis (Siegelin, Habel & Gaiser, 2008)
Extract (aerial parts)	*Sideritis scardica*	-	50 and 100 μg/mL	C6	Cytotoxicity and autophagy (Jeremic et al., 2013)
Extract (Njavara rice bran)	Kerala Agricultural University (Kerala India)	-	IC50 17,53 μg/mL (48 h)	C6	Antiproliferative effect (Rao et al., 2010)
Luteolin	Cayman Chemical Company (Ann Arbor, MI, USA)	https://pubchem.ncbi.nlm.nih.gov/compound/Luteolin	10, 15, 20 and 30 μM	U-87 MG T98-G	Inhibition of migration, downregulation of Cdc42, expression and PI3K/AKT activity (Cheng et al., 2013)
Quercetin	Sigma-Aldrich (St. Louis, MO, USA)	https://pubchem.ncbi.nlm.nih.gov/compound/Quercetin	25 to 100 μM	U-373 MG C6	Inhibition of cell proliferation and migration, increase in p53 expression, autophagy; cell death (mitochondrial pathway) (Kim et al., 2013; Da Silva et al., 2019)
Inflorescences of *Achyrocline satureioides*	3-O-metil-quercetin	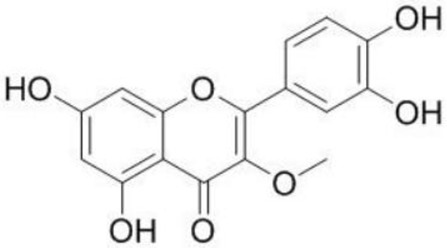	1 to 10 μM (72 h)	U-251 U-87 C6	Antiproliferative and pro-apoptotic effect (de Souza et al., 2018)
Rutin	Sigma-Aldrich (St. Louis, MO, USA)/ *Dimorphandra mollis Bent*	https://pubchem.ncbi.nlm.nih.gov/compound/Rutin	50 to 100 μM	GL-15	Reduction of proliferation and viabillity cells; apoptosis and astroglial differentiation (Santos et al., 2011)
Apigenin	Leaves of *Croton betulaster Müll.*	https://pubchem.ncbi.nlm.nih.gov/compound/Apigenin	50 and 100 μM	Microglial cells/C6	Antiproliferative and antimigratory effect, induction of cell differentiation, restoration of the immune function of the microglia, modification of the inflammatory profile (Coelho et al., 2019)
Licoflavone C (8-prenylapigenin)	Zhejiang University, (Hangzhou, China)	https://pubchem.ncbi.nlm.nih.gov/compound/Licoflavone-C	50 to 250 µmol/L	C6	Reduction of cell viability; Increase in caspase activity 3/7 (Wätjen et al., 2007)
Isobavachin (8- prenylliquiritigenin)	Zhejiang University, (Hangzhou, China)	https://pubchem.ncbi.nlm.nih.gov/compound/Isobavachin	50 to 250 µmol/L	C6	Reduction of cell viability; Increase in caspase activity 3/7 (Wätjen et al., 2007)
Hydroxygenkwanin	Y-J biological (Shanghai, China)	https://pubchem.ncbi.nlm.nih.gov/compound/5318214#section=2D-Structure	6.25 to 50 μM	C6	Antiproliferative effect, reduction of cell viability, loss of mitochondrial membrane potential, activation of caspase 3/8, negative expression of the BCL-XL, protein synergistic anti-glioma effect with apigenin (Wang et al., 2013)

* Internet references accessed at 30 October 2021.

**Table 2:** Some flavonoids that act in different signaling pathways in glioma cells.

Flavonoids	Effect in Models	Origin	Some Dietary Sources
**Luteolin** 3′, 4′, 5′, 7′- tetrahydroxyflavone	**I.** Inhibits the production of IL-6 in primary cultures of microglia from mice and in cultures of human BV-2 microglial cells, treated with 10, 25 and 50 μM and stimulated with LPS (Jang et al., 2008); I**I.** Over 15 μM, inhibited IL-1β release and downstream activation of NFκB in U-87glioblastoma cells culture (Lamy et al., 2014).	**I.** Purchased from Calbiochem and Synorex. I**I.** Purchased from Extrasynthese (Lyon, France).	Green pepper, parsley, chamomile (Jang et al., 2008). Green pepper, olive oil, parsley, celery, thyme, broccoli, cabbages and chamomile tea (Lamy et al., 2014).
**Quercetin** 2-(3,4-Dihydroxyphenyl)-3,5,7-trihydroxy-4H-chromen-4-one hydrate	**I.** Inhibits the IL-6 in T98G and U87 lineages treated with 25 μM (Michaud-Levesque et al., 2012); I**I.** Decreased the IL-8 levels in C6 cells culture (200 μM) in co-treatment with AG490, a JAK2 (Wang et al., 2013).	**I.** Not informed in the paper. I**I.** Not informed in the paper.	Red onion, apple, tea plant (Wang et al., 2013).
**Rutin** Quercetin-3-Rutinoside	**I.** In C6 lineage cells treated with 50 μM rutin, it reduced mRNA levels of L-1β, IL-6 and TNFα, induced a microglial polarization to M2 profile and increased IL-10 production after LPS stimulation (Silva et al., 2017).	**I.** Extracted from *Dimorphandra mollis* seeds.	Onions, apples, tea and red wine (Hosseinzadeh and Nassiri-Asl, 2014).
**Apigenin** 5,7-Dihydroxy-2-(4-hydroxyphenyl)-4H-chromen-4-one	**I.** Increases production and release of TNFα in C6 cell culture treated with 25.5, 25 and 50 μM apigenin (Wang et al., 2013).	**I.** Y-J biological (Shanghai, China).	Onions, celery, pistachio and burr parsley (Hollie, 2015).
**Fisetin** 3,7,3′,4′-tetrahydroxyflavone	**I.** Reduces ADAM9 expression in GBM8401 cells and inhibits the invasion of glioma after treatment with 20 and 40 μM (Chen et al., 2014).	**I.** Purchased from Sigma (St. Louis, MO, USA).	Strawberries, apples, onions, persimmons, grapes, wines, teas (Chen et al., 2014).
**Naringenin** 4′,5,7-Trihydroxyflavanone	**I.** Reduces COX2 activity in C6 glioma cells in a dose-dependent manner, impacting on tumor progression at 20 and 30 μg concentrations (Sabarinathan and Vanisree, 2011).	**I.** Purchased from Sigma.	Grapefruit and oranges (Sabarinathan and Vanisree, 2011).

## References

[1] Louis D.N., Perry A., Reifenberger G., von Deimling A., Figarella-Branger D., Cavenee W.K., Ohgaki H., Wiestler O.D., Kleihues P., Ellison D.W. (2016). The 2016 World Health Organization Classification of Tumors of the Central Nervous System: A summary. Acta Neuropathol..

[2] Davis M. (2016). Glioblastoma: Overview of Disease and Treatment. Clin. J. Oncol. Nurs..

[3] Li W., Graeber M.B. (2012). The molecular profile of microglia under the influence of glioma. Neuro-Oncology.

[4] Hosein Farzaei M., Bahramsoltani R., Rahimi R. (2016). Phytochemicals as Adjunctive with Conventional Anticancer Therapies. Curr. Pharm. Des.

[5] Santos B.L., Oliveira M.N., Coelho P.L.C., Pitanga B.P.S., da Silva A.B., Adelita T., Silva V.D.A., Costa M.d.D., El-Bachá R.S., Tardy M. (2015). Flavonoids suppress human glioblastoma cell growth by inhibiting cell metabolism, migration, and by regulating extracellular matrix proteins and metalloproteinases expression. Chem.-Biol. Interact.

[6] Coelho P.L., Oliveira M.N., da Silva A.B., Pitanga B.P., Silva V.D., Faria G.P., Sampaio G.P., Costa M.D.F.D., Braga-De-Souza S., Costa S.L. (2016). The flavonoid apigenin from Croton betulaster Mull inhibits proliferation, induces differentiation and regulates the inflammatory profile of glioma cells. Anti-Cancer Drugs.

[7] Wang J., Qi Q., Zhou W., Feng Z., Huang B., Chen A., Zhang D., Li W., Zhang Q., Jiang Z. (2018). Inhibition of glioma growth by flavokawain B is mediated through endoplasmic reticulum stress induced autophagy. Autophagy.

[8] Atiq A., Parhar I. (2020). Anti-neoplastic Potential of Flavonoids and Polysaccharide Phytochemicals in Glioblastoma. Molecules.

[9] Wu S.-Y., Watabe K. (2017). The roles of microglia macrophages in tumor progression of brain cancer and metastatic disease. Front. Biosci..

[10] da Silva A.B., Coelho P.L., das Neves Oliveira M., Oliveira J.L., Amparo J.A., da Silva K.C., Soares J.R., Pitanga B.P., dos Santos Souza C., de Faria Lopes G.P. (2020). The flavonoid rutin and its aglycone quercetin modulate the microglia inflammatory profile improving antiglioma activity. Brain, Behavior, and Immunity. Brain Behav. Immun..

[11] Coelho P.L., Amparo J.A.O., Da Silva A.B., Da Silva K.C., Braga-De-Souza S., Barbosa P.R., Lopes G.P.D.F., Costa S.L. (2019). Apigenin from *Croton betulaster Müll* restores the immune profile of microglia against glioma cells. Phytother. Res..

[12] Hanif F., Muzaffar K., Perveen K., Malhi S.M., Simjee S.U. (2017). Glioblastoma Multiforme: A Review of its Epidemiology and Pathogenesis through Clinical Presentation and Treatment. Asian Pac. J. Cancer Prev. APJCP.

[13] Batash R., Asna N., Schaffer P., Francis N., Schaffer M. (2017). Glioblastoma Multiforme, Diagnosis and Treatment; Recent Literature Review. Curr. Med. Chem..

[14] Friedmann-Morvinski D. (2014). Glioblastoma Heterogeneity and Cancer Cell Plasticity. Crit. Rev. Oncog..

[15] Gimple R.C., Bhargava S., Dixit D., Rich J.N. (2019). Glioblastoma stem cells: Lessons from the tumor hierarchy in a lethal cancer. Genes Dev..

[16] Lee J.H., Lee J.E., Kahng J.Y., Kim S.H., Park J.S., Yoon S.J., Um J.-Y., Kim W.K., Lee J.-K., Park J. (2018). Human glioblastoma arises from subventricular zone cells with low-level driver mutations. Nature.

[17] Yao M., Li S., Wu X., Diao S., Zhang G., He H., Bian L., Lu Y. (2018). Cellular origin of glioblastoma and its implication in precision therapy. Cell. Mol. Immunol..

[18] Wesseling P., Capper D. (2018). WHO 2016 Classification of gliomas. Neuropathol. Appl. Neurobiol..

[19] Choe G., Park J.K., Jouben-Steele L., Kremen T.J., Liau L.M., Vinters H.V., Cloughesy T.F., Mischel P.S. (2002). Active matrix metalloproteinase 9 expression is associated with primary glioblastoma subtype. Clin. Cancer Res..

[20] Ohgaki H., Kleihues P. (2007). Genetic Pathways to Primary and Secondary Glioblastoma. Am. J. Pathol..

[21] Alcantara Llaguno S.R., Parada L.F. (2016). Cell of origin of glioma: Biological and clinical implications. Br. J. Cancer.

[22] Soomro S.H., Ting L.R., Qing Y.Y., Ren M. (2017). Molecular biology of glioblastoma: Classification and mutational locations. J. Pak. Med. Assoc..

[23] Omuro A. (2013). Glioblastoma and Other Malignant Gliomas: A clinical review. JAMA.

[24] Cahill D., Turcan S. (2018). Origin of Gliomas. Semin. Neurol..

[25] Phillips H.S., Kharbanda S., Chen R., Forrest W.F., Soriano R.H., Wu T.D., Misra A., Nigro J.M., Colman H., Soroceanu L. (2006). Molecular subclasses of high-grade glioma predict prognosis, delineate a pattern of disease progression, and resemble stages in neurogenesis. Cancer Cell.

[26] Alexander B.M., Cloughesy T.F. (2017). Adult Glioblastoma. J. Clin. Oncol..

[27] Belda-Iniesta C., de Castro Carpeño J., Sáenz E.C., Guerrero P.C., Perona R., Barón M.G. (2006). Molecular biology of malignant gliomas. Clin. Transl. Oncol..

[28] Sanai N., Alvarez-Buylla A., Berger M.S. (2008). Neural Stem Cells and the Origin of Gliomas. N. Engl. J. Med..

[29] Matias D., Balça-Silva J., Da Graça G.C., Wanjiru C.M., Macharia L.W., Nascimento C.P., Roque N.R., Coelho-Aguiar J.M., Pereira C.M., Dos Santos M.F. (2018). Microglia/Astrocytes–Glioblastoma Crosstalk: Crucial Molecular Mechanisms and Microenvironmental Factors. Front. Cell. Neurosci..

[30] Irvin D.M., McNeill R.S., Bash R.E., Miller C.R. (2017). Intrinsic Astrocyte Heterogeneity Influences Tumor Growth in Glioma Mouse Models. Brain Pathol..

[31] Zheng W., Li Q., Zhao C., Da Y., Zhang H.-L., Chen Z. (2018). Differentiation of Glial Cells From hiPSCs: Potential Applications in Neurological Diseases and Cell Replacement Therapy. Front. Cell. Neurosci..

[32] Westphal M., Lamszus K. (2015). Circulating biomarkers for gliomas. Nat. Rev. Neurol..

[33] Zhao X., Chen R., Liu M., Feng J., Chen J., Hu K. (2017). Remodeling the blood–brain barrier microenvironment by natural products for brain tumor therapy. Acta Pharm. Sin. B.

[34] Kawashima T., Yashiro M., Kasashima H., Terakawa Y., Uda T., Nakajo K., Umaba R., Tanoue Y., Tamrakar S., Ohata K. (2019). Oligodendrocytes Up-regulate the Invasive Activity of Glioblastoma Cells via the Angiopoietin-2 Signaling Pathway. Anticancer. Res..

[35] Henrik Heiland D., Ravi V.M., Behringer S.P., Frenking J.H., Wurm J., Joseph K., Garrelfs N.W.C., Strähle J., Heynckes S., Grauvogel J. (2019). Tumor-associated reactive astrocytes aid the evolution of immunosuppressive environment in glioblastoma. Nat. Commun..

[36] Guan X., Hasan M.N., Maniar S., Jia W., Sun D. (2018). Reactive Astrocytes in Glioblastoma Multiforme. Mol. Neurobiol..

[37] Kim J.-K., Jin X., Sohn Y.-W., Jin X., Jeon H.-Y., Kim E.-J., Ham S.W., Jeon H.-M., Chang S.-Y., Oh S.-Y. (2014). Tumoral RANKL activates astrocytes that promote glioma cell invasion through cytokine signaling. Cancer Lett..

[38] Nieland L., Morsett L.M., Broekman M.L., Breakefield X.O., Abels E.R. (2021). Extracellular Vesicle-Mediated Bilateral Communication between Glioblastoma and Astrocytes. Trends Neurosci..

[39] Hallal S., Mallawaaratchy D.M., Wei H., Ebrahimkhani S., Stringer B.W., Day B.W., Boyd A.W., Guillemin G.J., Buckland M.E., Kaufman K.L. (2019). Extracellular Vesicles Released by Glioblastoma Cells Stimulate Normal Astrocytes to Acquire a Tumor-Supportive Phenotype Via p53 and MYC Signaling Pathways. Mol. Neurobiol..

[40] Buruiană A., Florian Ștefan I., Florian A.I., Timiș T.-L., Mihu C.M., Miclăuș M., Oșan S., Hrapșa I., Cataniciu R.C., Farcaș M. (2020). The Roles of miRNA in Glioblastoma Tumor Cell Communication: Diplomatic and Aggressive Negotiations. Int. J. Mol. Sci..

[41] Bhome R., Del Vecchio F., Lee G.-H., Bullock M.D., Primrose J., Sayan A.E., Mirnezami A.H. (2018). Exosomal microRNAs (exomiRs): Small molecules with a big role in cancer. Cancer Lett..

[42] Ma Q., Huang J.-T., Xiong Y.-G., Yang X.-Y., Han R., Zhu W.-W. (2017). MicroRNA-96 Regulates Apoptosis by Targeting PDCD4 in Human Glioma Cells. Technol. Cancer Res. Treat..

[43] Sousa C., Biber K., Michelucci A. (2017). Cellular and Molecular Characterization of Microglia: A Unique Immune Cell Population. Front. Immunol..

[44] Kettenmann H., Hanisch U.-K., Noda M., Verkhratsky A. (2011). Physiology of Microglia. Physiol. Rev..

[45] Poon C.C., Sarkar S., Yong V.W., Kelly J.J.P. (2017). Glioblastoma-associated microglia and macrophages: Targets for therapies to improve prognosis. Brain.

[46] Prionisti I., Bühler L.H., Walker P.R., Jolivet R.B. (2019). Harnessing Microglia and Macrophages for the Treatment of Glioblastoma. Front. Pharmacol..

[47] Couto M., Coelho-Santos V., Santos L., Fontes-Ribeiro C., Silva A.P., Gomes C.M.F. (2019). The interplay between glioblastoma and microglia cells leads to endothelial cell monolayer dysfunction via the interleukin-6-induced JAK2/STAT3 pathway. J. Cell. Physiol..

[48] Hide T., Komohara Y. (2020). Oligodendrocyte Progenitor Cells in the Tumor Microenvironment. Tumor Microenviron..

[49] Portela M., Venkataramani V., Fahey-Lozano N., Seco E., Losada-Perez M., Winkler F., Casas-Tintó S. (2019). Glioblastoma cells vampirize WNT from neurons and trigger a JNK/MMP signaling loop that enhances glioblastoma progression and neurodegeneration. PLoS Biol..

[50] Sattler R., Tyler B., Hoover B., Coddington L., Recinos V., Hwang L., Brem H., Rothstein J.D. (2013). Increased expression of glutamate transporter GLT-1 in peritumoral tissue associated with prolonged survival and decreases in tumor growth in a rat model of experimental malignant glioma. J. Neurosurg..

[51] Ribeiro M.P.C., Custódio J.B.A., Santos A.E. (2017). Ionotropic glutamate receptor antagonists and cancer therapy: Time to think out of the box?. Cancer Chemother. Pharmacol..

[52] Corsi L., Mescola A., Alessandrini A. (2019). Glutamate Receptors and Glioblastoma Multiforme: An Old “Route” for New Perspectives. Int. J. Mol. Sci..

[53] Cuddapah V.A., Robel S., Watkins S., Sontheimer H. (2014). A neurocentric perspective on glioma invasion. Nat. Rev. Neurosci..

[54] Maus A., Peters G.J. (2017). Glutamate and α-ketoglutarate: Key players in glioma metabolism. Amino Acids.

[55] Dirkse A., Golebiewska A., Buder T., Nazarov P.V., Muller A., Poovathingal S., Brons N.H.C., Leite S., Sauvageot N., Sarkisjan D. (2019). Stem cell-associated heterogeneity in Glioblastoma results from intrinsic tumor plasticity shaped by the microenvironment. Nat. Commun..

[56] Das A., Banik N.L., Ray S.K. (2009). Flavonoids activated caspases for apoptosis in human glioblastoma T98G and U87MG cells but not in human normal astrocytes. Cancer.

[57] Guo Z., Guozhang H., Wang H., Li Z., Liu N. (2019). Ampelopsin inhibits human glioma through inducing apoptosis and autophagy dependent on ROS generation and JNK pathway. Biomed. Pharmacother..

[58] Aroui S., Fetoui H., Kenani A. (2020). Natural dietary compound naringin inhibits glioblastoma cancer neoangiogenesis. BMC Pharmacol. Toxicol..

[59] Bever A.M., Cassidy A., Rimm E.B., Stampfer M.J., Cote D.J. (2021). A prospective study of dietary flavonoid intake and risk of glioma in US men and women. Am. J. Clin. Nutrition..

[60] Ho I.A.W., Shim W.S.N. (2017). Contribution of the Microenvironmental Niche to Glioblastoma Heterogeneity. BioMed Res. Int..

[61] Pavon L.F., Sibov T.T., Oliveira D., Marti L.C., Cabral F.R., De Souza J.G., Boufleur P., Malheiros S.M., Neto M.A.D.P., Da Cruz E.F. (2016). Mesenchymal stem cell-like properties of CD133+ glioblastoma initiating cells. Oncotarget.

[62] Atiya H., Frisbie L., Pressimone C., Coffman L. (2020). Mesenchymal Stem Cells in the Tumor Microenvironment. Tumor Microenviron..

[63] Schiffer D., Mellai M., Annovazzi L., Caldera V., Piazzi A., Denysenko T., Melcarne A. (2014). Stem Cell Niches in Glioblastoma: A Neuropathological View. BioMed Res. Int..

[64] Jhaveri N., Chen T.C., Hofman F.M. (2016). Tumor vasculature and glioma stem cells: Contributions to glioma progression. Cancer Lett..

[65] Ding D.-C., Shyu W.-C., Lin S.-Z. (2011). Mesenchymal Stem Cells. Cell Transplant..

[66] Kingery M.T., Manjunath A.K., Anil U., Strauss E.J. (2019). Bone Marrow Mesenchymal Stem Cell Therapy and Related Bone Marrow-Derived Orthobiologic Therapeutics. Curr. Rev. Musculoskelet. Med..

[67] Hashemi S.M., Hassan Z.M., Hossein-Khannazer N., Pourfathollah A.A., Soudi S. (2019). Investigating the route of administration and efficacy of adipose tissue-derived mesenchymal stem cells and conditioned medium in type 1 diabetic mice. Inflammopharmacology.

[68] Motegi S., Ishikawa O. (2017). Mesenchymal stem cells: The roles and functions in cutaneous wound healing and tumor growth. J. Dermatol. Sci..

[69] Sena I.F.G., Paiva A.E., Prazeres P.H.D.M., Azevedo P.O., Lousado L., Bhutia S.K., Salmina A.B., Mintz A., Birbrair A. (2018). Glioblastoma-activated pericytes support tumor growth via immunosuppression. Cancer Med..

[70] Xu C., Fu F., Li X., Zhang S. (2017). Mesenchymal stem cells maintain the microenvironment of central nervous system by regulating the polarization of macrophages/microglia after traumatic brain injury. Int. J. Neurosci..

[71] Li Z., Liu F., He X., Yang X., Shan F., Feng J. (2019). Exosomes derived from mesenchymal stem cells attenuate inflammation and demyelination of the central nervous system in EAE rats by regulating the polarization of microglia. Int. Immunopharmacol..

[72] Figueroa J., Phillips L.M., Shahar T., Hossain A., Gumin J., Kim H., Bean A.J., Calin G., Fueyo J., Walters E.T. (2017). Exosomes from Glioma-Associated Mesenchymal Stem Cells Increase the Tumorigenicity of Glioma Stem-like Cells via Transfer of miR-1587. Cancer Res..

[73] Hou L., Wang X., Zhou Y., Ma H., Wang Z., He J., Hu H., Guan W., Ma Y. (2013). Inhibitory effect and mechanism of mesenchymal stem cells on liver cancer cells. Tumor Biol..

[74] Dasari V.R., Kaur K., Velpula K.K., Gujrati M., Fassett D., Klopfenstein J.D., Dinh D.H., Rao J.S. (2010). Upregulation of PTEN in Glioma Cells by Cord Blood Mesenchymal Stem Cells Inhibits Migration via Downregulation of the PI3K/Akt Pathway. PLoS ONE.

[75] Nakamura K., Ito Y., Kawano Y., Kurozumi K., Kobune M., Tsuda H., Bizen A., Honmou O., Niitsu Y., Hamada H. (2004). Antitumor effect of genetically engineered mesenchymal stem cells in a rat glioma model. Gene Ther..

[76] Gomes E., de Castro J.V., Costa B., Salgado A. (2018). The impact of Mesenchymal Stem Cells and their secretome as a treatment for gliomas. Biochimie.

[77] Ridge S.M., Sullivan F.J., Glynn S.A. (2017). Mesenchymal stem cells: Key players in cancer progression. Mol. Cancer.

[78] Xu H., Zhao G., Zhang Y., Jiang H., Wang W., Zhao D., Hong J., Yu H., Qi L. (2019). Mesenchymal stem cell-derived exosomal microRNA-133b suppresses glioma progression via Wnt/β-catenin signaling pathway by targeting EZH2. Stem Cell Res. Ther..

[79] Amawi H., Ashby J.C.R., Samuel T., Peraman R., Tiwari A.K. (2017). Polyphenolic Nutrients in Cancer Chemoprevention and Metastasis: Role of the Epithelial-to-Mesenchymal (EMT) Pathway. Nutrients.

[80] Nascimento R.P., Santos B.L., Silva K.C., Amaral da Silva V.D., Fátima Costa M., David J.M., David J.P., Moura-Neto V., Oliveira M.d.N., Ulrich H. (2021). Reverted effect of mesenchymal stem cells in glioblastoma treated with agathisflavone and its selective antitumoral effect on cell viability, migration, and differentiation via STAT3. J. Cell. Physiol..

[81] Broekman M.L., Maas S.L.N., Abels E.R., Mempel T.R., Krichevsky A.M., Breakefield X.O. (2018). Multidimensional communication in the microenvirons of glioblastoma. Nat. Rev. Neurol..

[82] Formica J.V., Regelson W. (1995). Review of the biology of quercetin and related bioflavonoids. Food Chem. Toxicol..

[83] Panche A.N., Diwan A.D., Chandra S.R. (2016). Flavonoids: An overview. J. Nutr. Sci..

[84] Harwood M., Danielewska-Nikiel B., Borzelleca J.F., Flamm G.W., Williams G.M., Lines T.C. (2007). A critical review of the data related to the safety of quercetin and lack of evidence of in vivo toxicity, including lack of genotoxic/carcinogenic properties. Food Chem. Toxicol..

[85] Ravishankar D., Rajora A.K., Greco F., Osborn H. (2013). Flavonoids as prospective compounds for anti-cancer therapy. Int. J. Biochem. Cell Biol..

[86] D’Archivio M., Filesi C., di Benedetto R., Gargiulo R., Giovannini C., Masella R. (2007). Polyphenols, dietary sources and bioavailability. Ann. Dell’istituto Super. Sanita.

[87] Walle T., Browning A.M., Steed L.L., Reed S.G., Walle U.K. (2005). Flavonoid Glucosides Are Hydrolyzed and Thus Activated in the Oral Cavity in Humans. J. Nutr..

[88] Singla R.K., Dubey A.K., Garg A., Sharma R.K., Fiorino M., Ameen S.M., Haddad M.A., Al-Hiary M. (2019). Natural Polyphenols: Chemical Classification, Definition of Classes, Subcategories, and Structures. J. AOAC Int..

[89] Stalikas C.D. (2007). Extraction, separation, and detection methods for phenolic acids and flavonoids. J. Ofaration Sci..

[90] Havsteen B.H. (2002). The biochemistry and medical significance of the flavonoids. Pharmacol. Ther..

[91] Wang C.-Z., Li X.-L., Wang Q.-F., Mehendale S.R., Yuan C.-S. (2010). Selective fraction of Scutellaria baicalensis and its chemopreventive effects on MCF-7 human breast cancer cells. Phytomedicine.

[92] Birt D.F., Hendrich S., Wang W. (2001). Dietary agents in cancer prevention: Flavonoids and isoflavonoids. Pharmacol. Ther..

[93] Mink P.J., Scrafford C.G., Barraj L.M., Harnack L., Hong C.-P., Nettleton J.A., Jacobs D.R. (2007). Flavonoid intake and cardiovascular disease mortality: A prospective study in postmenopausal women. Am. J. Clin. Nutr..

[94] Faria A., Mateus N., Calhau C. (2012). Flavonoid transport across blood-brain barrier: Implication for their direct neuroprotective actions. Nutr. Aging.

[95] Hwang M.K., Song N.R., Kang N.J., Lee K.W., Lee H.J. (2009). Activation of phosphatidylinositol 3-kinase is required for tumor necrosis factor-α-induced upregulation of matrix metalloproteinase-9: Its direct inhibition by quercetin. Int. J. Biochem. Cell Biol..

[96] Amado N.G., Fonseca B.F., Cerqueira D.M., Neto V.M., Abreu J.G. (2011). Flavonoids: Potential Wnt/beta-catenin signaling modulators in cancer. Life Sci..

[97] Khan M., Yu B., Rasul A., al Shawi A., Yi F., Yang H., Ma T. (2012). Jaceosidin Induces Apoptosis in U87 Glioblastoma Cells through G2/M Phase Arrest. Evid.-Based Complementary Altern. Med..

[98] Liu K., Zhao F., Yan J., Xia Z., Jiang D., Ma P. (2020). Hispidulin: A promising flavonoid with diverse anti-cancer properties. Life Sci..

[99] Cheng W.-Y., Chiao M.-T., Liang Y.-J., Yang Y.-C., Shen C.-C., Yang C.-Y. (2013). Luteolin inhibits migration of human glioblastoma U-87 MG and T98G cells through downregulation of Cdc42 expression and PI3K/AKT activity. Mol. Biol. Rep..

[100] Chakrabarti M., Ray S.K. (2015). Anti-tumor activities of luteolin and silibinin in glioblastoma cells: Overexpression of miR-7-1-3p augmented luteolin and silibinin to inhibit autophagy and induce apoptosis in glioblastoma in vivo. Apoptosis.

[101] Balbinot R.B., De Oliveira J.A.M., Bernardi D.I., de Melo U.Z., Zanqueta B., Endo E.H., Ribeiro F.M., Volpato H., Figueiredo M.C., Back D.F. (2019). Structural Characterization and Biological Evaluation of 18-Nor-*ent*-labdane Diterpenoids from *Grazielia gaudichaudeana*. Chem. Biodivers..

[102] Mazimba O., Keroletswe N. (2015). Flavans: Synthetic Strategies: A Review. Int. Res. J. Pure Appl. Chem..

[103] Maués L., Alves G., Couto N., da Silva B., Arruda M., Macchi B., Sena C., Prado A., Crespo-Lopez M., Silva E. (2019). Flavonoids from the Amazon plant Brosimum acutifolium induce C6 glioma cell line apoptosis by disrupting mitochondrial membrane potential and reducing AKT phosphorylation. Biomed. Pharmacother..

[104] Reinli K., Block G. (1996). Phytoestrogen content of foods—A compendium of literature values. Nutr. Cancer.

[105] Křížová L., Dadáková K., Kašparovská J., Kašparovský T. (2019). Isoflavones. Molecules.

[106] Lei D., Zhang F., Yao D., Xiong N., Jiang X., Zhao H. (2018). Galangin increases ERK1/2 phosphorylation to decrease ADAM9 expression and prevents invasion in A172 glioma cells. Mol. Med. Rep..

[107] Siegelin M.D., Reuss D.E., Habel A., Herold-Mende C., von Deimling A. (2008). The flavonoid kaempferol sensitizes human glioma cells to TRAIL-mediated apoptosis by proteasomal degradation of survivin. Mol. Cancer Ther..

[108] Kong Y., Feng Z., Chen A., Qi Q., Han M., Wang S., Zhang Y., Zhang X., Yang N., Wang J. (2019). The Natural Flavonoid Galangin Elicits Apoptosis, Pyroptosis, and Autophagy in Glioblastoma. Front. Oncol..

[109] Guha A., Feldkamp M.M., Lau N., Boss G., Pawson A. (1997). Proliferation of human malignant astrocytomas is dependent on Ras activation. Oncogene.

[110] Lo H.-W. (2010). Targeting Ras-RAF-ERK and its Interactive Pathways as ael Therapy for Malignant Gliomas. Curr. Cancer Drug Targets.

[111] Kim H., Moon J.Y., Ahn K.S., Cho S.K. (2013). Quercetin Induces Mitochondrial Mediated Apoptosis and Protective Autophagy in Human Glioblastoma U373MG Cells. Oxidative Med. Cell. Longev..

[112] Bi Y., Shen C., Li C., Liu Y., Gao D., Shi C., Peng F., Liu Z., Zhao B., Zheng Z. (2016). Inhibition of autophagy induced by quercetin at a late stage enhances cytotoxic effects on glioma cells. Tumor Biol..

[113] Pan H.-C., Jiang Q., Yu Y., Mei J.-P., Cui Y.-K., Zhao W.-J. (2015). Quercetin promotes cell apoptosis and inhibits the expression of MMP-9 and fibronectin via the AKT and ERK signalling pathways in human glioma cells. Neurochem. Int..

[114] Park M.H., Min D.S. (2011). Quercetin-induced downregulation of phospholipase D1 inhibits proliferation and invasion in U87 glioma cells. Biochem. Biophys. Res. Commun..

[115] Marusawa H., Matsuzawa S., Welsh K., Zou H., Armstrong R., Tamm I., Reed J.C. (2003). HBXIP functions as a cofactor of survivin in apoptosis suppression. EMBO J..

[116] Fan X., Wang Y., Zhang C., Liu L., Yang S., Wang Y., Liu X., Qian Z., Fang S., Qiao H. (2016). ADAM9 Expression Is Associate with Glioma Tumor Grade and Histological Type, and Acts as a Prognostic Factor in Lower-Grade Gliomas. Int. J. Mol. Sci..

[117] Chen C.-M., Hsieh Y.-H., Hwang J.-M., Jan J.-H., Hsieh S.-C., Lin S.-H., Lai C.-Y. (2015). Fisetin suppresses ADAM9 expression and inhibits invasion of glioma cancer cells through increased phosphorylation of ERK1/2. Tumor Biol..

[118] Santos B.L., Silva A.R., Pitanga B.P.S., Sousa C.S., Grangeiro M.S., Fragomeni B.O., Coelho P.L.C., Oliveir M.N., Menezes-Filho N.J., Costa M.F.D. (2011). Antiproliferative, proapoptotic and morphogenic effects of the flavonoid rutin on human glioblastoma cells. Food Chem..

[119] Quan K., Zhang X., Fan K., Liu P., Yue Q., Li B., Wu J., Liu B., Xu Y., Hua W. (2017). Icariside II induces cell cycle arrest and apoptosis in human glioblastoma cells through suppressing Akt activation and potentiating FOXO3a activity. Am. J. Transl. Res..

[120] de Pascual-Teresa S., Moreno D.A., García-Viguera C. (2010). Flavanols and Anthocyanins in Cardiovascular Health: A Review of Current Evidence. Int. J. Mol. Sci..

[121] Manach C., Williamson G., Morand C., Scalbert A., Rémésy C. (2008). Bioavailability and bioefficacy of polyphenols in humans. I. Review of 97 bioavailability studies. Am. J. Clin. Nutr..

[122] Siddiqui I.A., Malik A., Adhami V.M., Asim M., Hafeez B.B., Sarfaraz S., Mukhtar H. (2008). Green tea polyphenol EGCG sensitizes human prostate carcinoma LNCaP cells to TRAIL-mediated apoptosis and synergistically inhibits biomarkers associated with angiogenesis and metastasis. Oncogene.

[123] Althagafy H.S., Meza-Aviña M.E., Oberlies N.H., Croatt M.P. (2013). Mechanistic Study of the Biomimetic Synthesis of Flavonolignan Diastereoisomers in Milk Thistle. J. Org. Chem..

[124] Crocenzi F., Roma M. (2006). Silymarin as a New Hepatoprotective Agent in Experimental Cholestasis: New Possibilities for an Ancient Medication. Curr. Med. Chem..

[125] Son Y.-G., Kim E., Kim J.Y., Kim S.U., Kwon T.K., Yoon A.-R., Yun C.-O., Choi K.S. (2007). Silibinin sensitizes human glioma cells to TRAIL-mediated apoptosis via DR5 up-regulation and down-regulation of c-FLIP and survivin. Cancer Res..

[126] Kim K.W., Choi C.H., Kim T.H., Kwon C.H., Woo J.S., Kim Y.K. (2009). Silibinin Inhibits Glioma Cell Proliferation via Ca^2+^/ROS/MAPK-Dependent Mechanism In Vitro and Glioma Tumor Growth In Vivo. Neurochem. Res..

[127] Jeong J.C., Shin W.Y., Kim T.H., Kwon C.H., Kim J.H., Kim Y.K., Kim K.H. (2011). Silibinin induces apoptosis via calpain-dependent AIF nuclear translocation in U87MG human glioma cell death. J. Exp. Clin. Cancer Res..

[128] Zhang M., Liu Y., Gao Y., Li S. (2015). Silibinin-induced glioma cell apoptosis by PI3K-mediated but Akt-independent downregulation of FoxM1 expression. Eur. J. Pharmacol..

[129] Zhen Z.G., Ren S.H., Ji H.M., Ma J.H., Ding X.M., Feng F.Q., Chen S.L., Zou P., Ren J.R., Jia L. (2017). Linarin suppresses glioma through inhibition of NF-κB/p65 and up-regulating p53 expression in vitro and in vivo. Biomed. Pharmacother..

[130] Yoshigai E., Machida T., Okuyama T., Mori M., Murase H., Yamanishi R., Okumura T., Ikeya Y., Nishino H., Nishizawa M. (2013). Citrus nobiletin suppresses inducible nitric oxide synthase gene expression in interleukin-1β-treated hepatocytes. Biochem. Biophys. Res. Commun..

[131] Zhang L., Zhang X., Zhang C., Bai X., Zhang J., Zhao X., Chen L., Wang L., Zhu C., Cui L. (2016). Nobiletin promotes antioxidant and anti-inflammatory responses and elicits protection against ischemic stroke in vivo. Brain Res..

[132] Nakajima A., Aoyama Y., Shin E.J., Nam Y., Kim H.C., Nagai T., Yokosuka A., Mimaki Y., Yokoi T., Ohizumi Y. (2015). Nobiletin, a citrus flavonoid, improves cognitive impairment and reduces soluble Aβ levels in a triple transgenic mouse model of Alzheimer’s disease (3XTg-AD). Behav. Brain Res..

[133] Aoki K., Yokosuka A., Mimaki Y., Fukunaga K., Yamakuni T. (2013). Nobiletin Induces Inhibitions of Ras Activity and Mitogen-Activated Protein Kinase Kinase/Extracellular Signal-Regulated Kinase Signaling to Suppress Cell Proliferation in C6 Rat Glioma Cells. Biol. Pharm. Bull..

[134] Zhang L., Wang H., Cong Z., Xu J., Zhu J., Ji X., Ding K. (2014). Wogonoside induces autophagy-related apoptosis in human glioblastoma cells. Oncol. Rep..

[135] Zou M., Hu C., You Q., Zhang A., Wang X., Guo Q. (2015). Oroxylin A induces autophagy in human malignant glioma cells via the mTOR-STAT3-Notch signaling pathway. Mol. Carcinog..

[136] Coelho P.L.C., De Freitas S.R.V.-B., Pitanga B.P.S., Da Silva V.D.A., Oliveira M.N., Grangeiro M.S., Souza C., El-Bachá R.D.S., Costa M.D.F.D., Barbosa P.R. (2016). Flavonoids from the Brazilian plant Croton betulaster inhibit the growth of human glioblastoma cells and induce apoptosis. Rev. Bras. Farmacogn..

[137] Wang G., Wang J.J., Chen X.L., Du S.M., Li D.S., Pei Z.J., Lan H., Wu L.B. (2013). The JAK2/STAT3 and mitochondrial pathways are essential for quercetin nanoliposome-induced C6 glioma cell death. Cell Death Dis..

[138] Wätjen W., Weber N., Lou Y.J., Wang Z.Q., Chovolou Y., Kampkötter A., Kahl R., Proksch P. (2007). Prenylation enhances cytotoxicity of apigenin and liquiritigenin in rat H4IIE hepatoma and C6 glioma cells. Food Chem. Toxicol..

[139] Erdman J.W., Balentine D., Arab L., Beecher G., Dwyer J.T., Folts J., Harnly J., Hollman P., Keen C.L., Mazza G. (2007). Flavonoids and heart health: Proceedings of the ILSI North America flavonoids workshop, May 31–June 1, 2005, Washington, DC. J. Nutr..

[140] Engelhardt U.H., Finger A., Kuhr S. (1993). Determination of flavone C-glycosides in tea. Z. Lebensm.-Unters. Forsch..

[141] Gambelli L., Santaroni G.P. (2004). Polyphenols content in some Italian red wines of different geographical origins. J. Food Compos. Anal..

[142] Caristi C., Bellocco E., Gargiulli C., Toscano G., Leuzzi U. (2006). Flavone-di--glycosides in juices from Southern Italy. Food Chem..

[143] Lechtenberg M., Zumdick S., Gerhards C., Schmidt T.J., Hensel A. (2007). Evaluation of analytical markers characterising different drying methods of parsley leaves (*Petroselinum crispum* L.). Die Pharm..

[144] Sun Y., Fang N., Chen D.D.Y., Donkor K.K. (2008). Determination of potentially anti-carcinogenic flavonoids in wines by micellar electrokinetic chromatography. Food Chem..

[145] Pereira-Caro G., Cros G., Yokota T., Crozier A. (2013). Phytochemical Profiles of Black, Red, Brown, and White Rice from the Camargue Region of France. J. Agric. Food Chem..

[146] Kreft I., Zhou M., Golob A., Germ M., Likar M., Dziedzic K., Luthar Z. (2020). Breeding buckwheat for nutritional quality. Breed. Sci..

[147] Chen T.-J., Jeng J.-Y., Lin C.-W., Wu C.-Y., Chen Y.-C. (2006). Quercetin inhibition of ROS-dependent and-independent apoptosis in rat glioma C6 cells. Toxicology.

[148] Freitas S., Costa S., Azevedo C., Carvalho G., Freire S., Barbosa P., Velozo E., Schaer R., Tardy M., Meyer R. (2010). Flavonoids inhibit angiogenic cytokine production by human glioma cells. Phytother. Res..

[149] Silva A.R., Pinheiro A.M., Souza C.S., Freitas S.R.V.-B., Vasconcellos V., Freire S.M., Velozo E.S., Tardy M., El-Bachá R., Costa M.F.D. (2008). The flavonoid rutin induces astrocyte and microglia activation and regulates TNF-alpha and NO release in primary glial cell cultures. Cell Biol. Toxicol..

[150] Barreca D., Gattuso G., Bellocco E.S., Calderaro A., Trombetta D., Smeriglio A., Laganà G., Daglia M., Meneghini S., Nabavi S.M. (2017). Flavanones: Citrus phytochemical with health-promoting properties. BioFactors.

[151] Stompor M., Uram Ł., Podgórski R. (2017). In Vitro Effect of 8-Prenylnaringenin and Naringenin on Fibroblasts and Glioblastoma Cells-Cellular Accumulation and Cytotoxicity. Molecules.

[152] Aroui S., Najlaoui F., Chtourou Y., Meunier A.-C., Laajimi A., Kenani A., Fetoui H. (2016). Naringin inhibits the invasion and migration of human glioblastoma cell via downregulation of MMP-2 and MMP-9 expression and inactivation of p38 signaling pathway. Tumor Biol..

[153] Ono K., Han J. (1999). The p38 signal transduction pathway Activation and function. Cell. Signal..

[154] Ferreira A., Rodrigues M., Fortuna A., Falcão A., Alves G. (2018). Flavonoid compounds as reversing agents of the P-glycoprotein-mediated multidrug resistance: An in vitro evaluation with focus on antiepileptic drugs. Food Res. Int..

[155] Rammohan A., Reddy J.S., Sravya G., Rao C.N., Zyryanov G.V. (2020). Chalcone synthesis, properties and medicinal applications: A review. Environ. Chem. Lett..

[156] Zhou G.-S., Song L.-J., Yang B. (2013). Isoliquiritigenin inhibits proliferation and induces apoptosis of U87 human glioma cells in vitro. Mol. Med. Rep..

[157] Lin Y., Sun H., Dang Y., Li Z. (2017). Isoliquiritigenin inhibits the proliferation and induces the differentiation of human glioma stem cells. Oncol. Rep..

[158] Deharo E., Ginsburg H. (2011). Analysis of additivity and synergism in the anti-plasmodial effect of purified compounds from plant extracts. Malar. J..

[159] Jeremic I., Tadic V., Isakovic A., Trajkovic V., Markovic I., Redzic Z., Isakovic A. (2013). The Mechanisms of In Vitro Cytotoxicity of Mountain Tea, Sideritis scardica, against the C6 Glioma Cell Line. Planta Med..

[160] Rao A.S., Reddy S.G., Babu P.P., Reddy A.R. (2010). The antioxidant and antiproliferative activities of methanolic extracts from Njavara rice bran. BMC Complementary Altern. Med..

[161] De Souza P.O., Bianchi S.E., Figueiró F., Heimfarth L., Moresco K.S., Gonçalves R.M., Hoppe J.B., Klein C.P., Salbego C.G., Gelain D.P. (2018). Anticancer activity of flavonoids isolated from Achyrocline satureioides in gliomas cell lines. Toxicol. Vitro.

[162] Dell’Albani P., dico B., Grasso S., Rocco C., Foti M.C. (2017). Quercetin derivatives as potent inducers of selective cytotoxicity in glioma cells. Eur. J. Pharm. Sci..

[163] Rivero-Cruz J.F., Granados-Pineda J., Pedraza-Chaverri J., Pérez-Rojas J.M., Kumar-Passari A., Diaz-Ruiz G., Rivero-Cruz B.E. (2020). Phytochemical Constituents, Antioxidant, Cytotoxic, and Antimicrobial Activities of the Ethanolic Extract of Mexican Brown Propolis. Antioxidants.

[164] Ahmad F., Seerangan P., Mustafa M.Z., Osman Z.F., Abdullah J.M., Idris Z. (2019). Anti-Cancer Properties of Heterotrigona itama sp. Honey Via Induction of Apoptosis in Malignant Glioma Cells. Malays. J. Med Sci..

[165] Tai M.C., Tsang S.Y., Chang L.Y.F., Xue H. (2006). Therapeutic Potential of Wogonin: A Naturally Occurring Flavonoid. CNS Drug Rev..

[166] Li C., Lin G., Zuo Z. (2011). Pharmacological effects and pharmacokinetics properties of Radix Scutellariae and its bioactive flavones. Biopharm. Drug Dispos..

[167] Parajuli P., Joshee N., Rimando A., Mittal S., Yadav A. (2009). In vitro Antitumor Mechanisms of Various *Scutellaria* Extracts and Constituent Flavonoids. Planta Med..

[168] Yamaguchi S., Kobayashi H., Terasaka S., Ishii N., Ikeda J., Kanno H., Nishihara H., Tanaka S., Houkin K. (2012). The Impact of Extent of Resection and Histological Subtype on the Outcome of Adult Patients with High-grade Gliomas. Jpn. J. Clin. Oncol..

[169] Anjum K., Shagufta B.I., Abbas S.Q., Patel S., Khan I., Shah S.A.A., Akhter N., Hassan S.S.U. (2017). Current status and future therapeutic perspectives of glioblastoma multiforme (GBM) therapy: A review. Biomed. Pharmacother..

[170] Stupp R., Taillibert S., Kanner A.A., Kesari S., Steinberg D.M., Toms S.A., Taylor L.P., Liewberman F., Silvani A., Fink K.L. (2017). Effect of tumor-treating fields plus maintenance temozolomide vs maintenance temozolomide alone on survival in patients with glioblastoma: A randomized clinical trial. JAMA.

[171] Agarwala S.S., Kirkwood J.M. (2000). Temozolomide, ael Alkylating Agent with Activity in the Central Nervous System, Improve the Treatment of Advanced Metastatic Melanoma. Oncologist.

[172] Strobel H., Baisch T., Fitzel R., Schilberg K., Siegelin M.D., Karpel-Massler G., Debatin K.-M., Westhoff M.-A. (2019). Temozolomide and Other Alkylating Agents in Glioblastoma Therapy. Biomedicines.

[173] Desai V., Jain A., Shaghaghi H., Summer R., Lai J.C.K., Bhushan A. (2019). Combination of Biochanin A and Temozolomide Impairs Tumor Growth by Modulating Cell Metabolism in Glioblastoma Multiforme. Anticancer. Res..

[174] Wang C., Chen Y., Wang Y., Liu X., Liu Y., Li Y., Chen H., Fan C., Wu D., Yang J. (2019). Inhibition of COX-2, mPGES-1 and CYP4A by isoliquiritigenin blocks the angiogenic Akt signaling in glioma through ceRNA effect of miR-194-5p and lncRNA NEAT1. J. Exp. Clin. Cancer Res..

[175] Sharma V., Joseph C., Ghosh S., Agarwal A., Mishra M.K., Sen E. (2007). Kaempferol induces apoptosis in glioblastoma cells through oxidative stress. Mol. Cancer Ther..

[176] Dizaji M.Z., Malehmir M., Ghavamzadeh A., Alimoghaddam K., Ghaffari S.H. (2012). Synergistic Effects of Arsenic Trioxide and Silibinin on Apoptosis and Invasion in Human Glioblastoma U87MG Cell Line. Neurochem. Res..

[177] Yang I., Han S.J., Kaur G., Crane C., Parsa A.T. (2010). The role of microglia in central nervous system immunity and glioma immunology. J. Clin. Neurosci..

[178] Marumoto T., Saya H. (2012). Molecular Biology of Glioma. Glioma.

[179] Hermansen S.K., Nielsen B.S., Aaberg-Jessen C., Kristensen B.W. (2016). Mir-21 Is Linked to Glioma Angiogenesis. J. Histochem. Cytochem..

[180] Ferrer V.P., Moura Neto V., Mentlein R. (2018). Glioma infiltration and extracellular matrix: Key players and modulators. Glia.

[181] Yang Y.-C., Chiou P.-C., Chen P.-C., Liu P.-Y., Huang W.-C., Chao C.-C., Tang C.-H. (2019). Melatonin reduces lung cancer stemness through inhibiting of PLC, ERK, p38, β-catenin, and Twist pathways. Environ. Toxicol.

[182] Schindler R., Mentlein R. (2006). Flavonoids and Vitamin E Reduce the Release of the Angiogenic Peptide Vascular Endothelial Growth Factor from Human Tumor Cells. J. Nutr..

[183] Lien L.-M., Wang M.-J., Chen R.-J., Chiu H.-C., Wu J.-L., Shen M.-Y., Chou D.-S., Sheu J.-R., Lin K.-H., Lu W.-J. (2016). Nobiletin, a Polymethoxylated Flavone, Inhibits Glioma Cell Growth and Migration via Arresting Cell Cycle and Suppressing MAPK and Akt Pathways. Phytother. Res..

[184] Hambardzumyan D., Gutmann D.H., Kettenmann H. (2016). The role of microglia and macrophages in glioma maintenance and progression. Nat. Neurosci..

[185] Holtman I.R., Skola D., Glass C.K. (2017). Transcriptional control of microglia phenotypes in health and disease. J. Clin. Investig..

[186] Tang Y., Le W. (2016). Differential Roles of M1 and M2 Microglia in Neurodegenerative Diseases. Mol. Neurobiol..

[187] Komohara Y., Ohnishi K., Kuratsu J., Takeya M. (2008). Possible involvement of the M2 anti-inflammatory macrophage phenotype in growth of human gliomas. J. Pathol..

[188] Laudati E., Currò D., Navarra P., Lisi L. (2017). Blockade of CCR5 receptor prevents M2 microglia phenotype in a microglia-glioma paradigm. Neurochem. Int..

[189] Mostofa A.G.M., Punganuru S.R., Madala H.R., Al-Obaide M., Srivenugopal K.S. (2017). The Process and Regulatory Components of Inflammation in Brain Oncogenesis. Biomolecules.

[190] Ferguson S.D., Srinivasan V.M., Heimberger A.B. (2015). The role of STAT3 in tumor-mediated immune suppression. J. Neuro-Oncol..

[191] Luwor R.B., Stylli S.S., Kaye A.H. (2013). The role of Stat3 in glioblastoma multiforme. J. Clin. Neurosci..

[192] Michaud-Levesque J., Bousquet-Gagnon N., Béliveau R. (2013). Quercetin abrogates IL-6/STAT3 signaling and inhibits glioblastoma cell line growth and migration. Exp. Cell Res..

[193] Goswami S., Gupta A., Sharma S.K. (2002). Interleukin-6-Mediated Autocrine Growth Promotion in Human Glioblastoma Multiforme Cell Line U87MG. J. Neurochem..

[194] Li R., Li G., Deng L., Liu Q., Dai J., Shen J., Zhang J. (2010). IL-6 augments the invasiveness of U87MG human glioblastoma multiforme cells via up-regulation of MMP-2 and fascin-1. Oncol. Rep..

[195] Jang S., Kelley K.W., Johnson R.W. (2008). Luteolin reduces IL-6 production in microglia by inhibiting JNK phosphorylation and activation of AP-1. Proc. Natl. Acad. Sci. USA.

[196] Lamy S., Moldovan P.L., ben Saad A., Annabi B. (2015). Biphasic effects of luteolin on interleukin-1β-induced cyclooxygenase-2 expression in glioblastoma cells. Biochim. Biophys. Acta-Mol. Cell Res..

[197] Sharma V., Dixit D., Koul N., Mehta V.S., Sen E. (2011). Ras regulates interleukin-1β-induced HIF-1α transcriptional activity in glioblastoma. J. Mol. Med..

[198] Yeung Y., McDonald K., Grewal T., Munoz L. (2013). Interleukins in glioblastoma pathophysiology: Implications for therapy. Br. J. Pharmacol..

[199] Xia L., Tan S., Zhou Y., Lin J., Wang H., Oyang L., Tian Y., Liu L., Su M., Wang H. (2018). Role of the NFκB-signaling pathway in cancer. Onco. Targets Ther..

[200] Sun Y., Liu W.-Z., Liu T., Feng X., Yang N., Zhou H.-F. (2015). Signaling pathway of MAPK/ERK in cell proliferation, differentiation, migration, senescence and apoptosis. J. Recept. Signal Transduct..

[201] Fathima Hurmath K., Ramaswamy P., Nandakumar D.N. (2014). IL-1β microenvironment promotes proliferation, migration, and invasion of human glioma cells. Cell Biol. Int..

[202] Minchenko O.H., Tsymbal D.O., Minchenko D.O., Ratushna O.O. (2016). The role of the TNF receptors and apoptosis inducing ligands in tumor growth. Ukr. Biochem. J..

[203] Kashyap D., Garg V.K., Tuli H.S., Yerer M.B., Sak K., Sharma A.K., Kumar M., Aggarwal V., Sandhu S.S. (2019). Fisetin and Quercetin: Promising Flavonoids with Chemopreventive Potential. Biomolecules.

[204] Zeng W., Jin L., Zhang F., Zhang C., Liang W. (2018). Naringenin as a potential immunomodulator in therapeutics. Pharmacol. Res..

[205] Imran M., Rauf A., Abu-Izneid T., Nadeem M., Shariati M.A., Khan I.A., Imran A., Orhan I.E., Rizwan M., Atif M. (2019). Luteolin, a flavonoid, as an anticancer agent: A review. Biomed. Pharmacother..

[206] Kamran N., Alghamri M.S., Nunez F.J., Shah D., Asad A.S., Candolfi M., Altshuler D., Lowenstein P.R., Castro M.G. (2018). Current state and future prospects of immunotherapy for glioma. Immunotherapy.

[207] Malik S., Suchal K., Khan S.I., Bhatia J., Kishore K., Dinda A.K., Arya D.S. (2017). Apigenin ameliorates streptozotocin-induced diabetic nephropathy in rats via MAPK-NF-κB-TNF-α and TGF-β1-MAPK-fibronectin pathways. Am. J. Physiol.-Ren. Physiol..

[208] Muñoz-Pinedo C. (2012). Signaling Pathways that Regulate Life and Cell Death: Evolution of Apoptosis in the Context of Self-Defense. Self Nonself.

[209] Salmaggi A., Eoli M., Frigerio S., Silvani A., Gelati M., Corsini E., Broggi G., Boiardi A. (2003). Intracavitary VEGF, bFGF, IL-8, IL-12 levels in primary and recurrent malignant glioma. J. Neuro-Oncol..

[210] Hong T.-M., Teng L.-J., Shun C.-T., Peng M.-C., Tsai J.-C. (2009). Induced interleukin-8 expression in gliomas by tumor-associated macrophages. J. Neuro-Oncol..

[211] Yang P.-C., Yuan A., Chen J.J.W., Yao P.-L. (2005). The role of interleukin-8 in cancer cells and microenvironment interaction. Front. Biosci..

[212] Fabbri E., Brognara E., Montagner G., Ghimenton C., Eccher A., Cantù C., Khalil S., Bezzerri V., Provezza L., Bianchi N. (2015). Regulation of IL-8 gene expression in gliomas by microRNA miR-93. BMC Cancer.

[213] Couper K.N., Blount D.G., Riley E.M. (2008). IL-10: The Master Regulator of Immunity to Infection. J. Immunol..

[214] Saraiva M., O’Garra A. (2010). The regulation of IL-10 production by immune cells. Nat. Rev. Immunol..

[215] Ji J.D., Kim H.J., Rho Y.H., Choi S.J., Lee Y.H., Cheon H.J., Sohn J., Song G.G. (2008). Inhibition of IL-10-induced STAT3 activation by 15-deoxy-Δ12,14-prostaglandin J_2_. Rheumatology.

